# Hybrid Care Modifications in the Delivery of Nonpandemic Care During the COVID-19 Pandemic: Scoping Review

**DOI:** 10.2196/84756

**Published:** 2026-04-30

**Authors:** Natalia Sanchez Villalobos, Tessa van Loenen, Lem Ngongalah, Máire A Connolly, Mart L Stein, Chantal P Rovers, Aura Timen

**Affiliations:** 1 Department of Primary and Community Care, Radboud University Medical Center, Geert Grooteplein Noord 21, Nijmegen, 6525EZ, The Netherlands, 31 0243618181; 2 School of Health Sciences, University of Galway, Galway, Ireland; 3 Centre for Infectious Disease Control, National Institute for Public Health and the Environment, Bilthoven, The Netherlands; 4 Department of Internal Medicine, Radboud University Medical Center, Nijmegen, The Netherlands

**Keywords:** hybrid care, digital health, telemedicine, COVID-19 pandemic, health care delivery

## Abstract

**Background:**

The COVID-19 pandemic had an unprecedented impact on the delivery of health care, with digital interventions accelerating more than ever before. However, evidence of how hybrid care models, combining digital health interventions with in-person care, were implemented during the pandemic remains scattered. Understanding hybrid care models is imperative to build resilient health systems that can ensure access to care during crisis situations.

**Objective:**

The study aimed to examine the implementation of hybrid care modifications to support the delivery of nonpandemic health care services in Europe during the COVID-19 pandemic.

**Methods:**

A scoping review was conducted following PRISMA-ScR (Preferred Reporting Items for Systematic Reviews and Meta-Analyses Extension for Scoping Reviews) guidelines. Systematic searches were conducted in PubMed or MEDLINE, Embase, CINAHL, Web of Science, and PsycINFO on May 22, 2024, and updated on January 14, 2026. Studies were eligible if they included primary data on the use of digital care modifications implemented or scaled up during the COVID-19 pandemic for the delivery of nonpandemic health care services in Europe. Non–peer-reviewed publications and studies with a primary focus on mental health or pediatric care were excluded. Quality appraisal was conducted using the Mixed Methods Appraisal Tool. Descriptions of digital care modifications were inductively analyzed and used to create digital flows, combining telehealth systems, digital interventions, and care functions. Digital care modifications were categorized according to their hybrid care implementation (digital-only or hybrid). Study evaluations were extracted using the Kirkpatrick model.

**Results:**

A total of 189 studies were included for analysis. Studies covered evidence from 2020 to 2024, a total of 23 countries, and 37 health care disciplines. Hybrid care implementation was reported in over 60% (115/189) of the studies, describing various forms of digital and in-person care. Care modifications incorporating in-person and digital care components were more commonly described in specialty care contexts. A total of 68 distinct digital flows were identified, with a limited number of telehealth systems allowing substantial variety in both interventions and care functions. Prominent digital flows included the use of online platforms to support video and messaging for follow-up care. Over half of the studies did not describe any kind of evaluation.

**Conclusions:**

This review has shown how few telehealth systems were able to support a variety of care functions in the delivery of nonpandemic care throughout the COVID-19 pandemic, underscoring their practical versatility. Integrating digital health as part of hybrid care models is essential in designing care pathways that can adapt to different contexts, including future health crises. Although a comprehensive search was conducted, the heterogeneous reporting of care modifications may have influenced the interpretation of the findings. In the future, research may expand the application of hybrid care models to innovative strategies for effective crisis management.

## Introduction

The COVID-19 pandemic had an unprecedented impact on the delivery of health care, leading to modifications that sought to compensate for service disruptions. By 2022, the World Health Organization reported that over 90% of countries worldwide were still facing disruptions in health service delivery [1]. As health systems continue to recover post pandemic, it is critical to understand the modifications implemented to support the delivery of nonpandemic health care (ie, health care provided for conditions other than COVID-19 disease). Among the various modifications implemented, the rapid surge in the use of digital health played a critical role in responding to the challenges posed by the pandemic, which restricted resources and impeded the safe delivery of in-person care [2]. Understanding modifications to care, particularly those involving digital health, is crucial for building resilient health systems that can ensure access to care, including during emergency situations.

Digital health refers to the use of technologies, including those for communication and information exchange, to support health systems [3]. Previous literature has covered the adoption and use of digital health during the COVID-19 pandemic, showing its potential benefits for operational and clinical applications such as data services, population-level responses, decision-support systems, and direct patient care [4,5]. For instance, the value of digital health interventions during the pandemic has been described for patients with conditions such as cancer and heart failure [6,7]. Although it has been recognized that digital health should be examined beyond the implementation of individual tools, research to date has tended to focus on standalone digital health interventions. Rather than studying these interventions as silos, they can be better examined as part of broader care pathways [5], highlighting the critical need for integration into existing health care systems [8].

Alongside digital health interventions, hybrid approaches have emerged as integrated alternatives to ensure the continuity of essential health services. Hybrid care can be defined as the delivery of health care services involving in-person (face-to-face) care and digital health components. Hybrid care provision has been studied previously in various contexts [9-12], including explorations of its potential in contrast to standard models of care for patients with chronic conditions [13,14]. In the context of the COVID-19 pandemic, previous research has shown a positive impact of the use of hybrid care models in specific clinical contexts [15]. Additionally, research on the effectiveness of telehealth compared to in-person care has highlighted the importance of further researching the integration between these types of care [16].

Given the increased uptake, hybrid care is anticipated to become the new norm for health care delivery in the coming years, offering novel avenues for unlocking access to care during regular and crisis situations. However, little is still known about how, and for which purposes, these models of care have been implemented and evaluated in previous crisis contexts. A scoping review was chosen to systematically map available evidence regarding the different types of modifications to care. This scoping review aimed to examine the implementation of hybrid care modifications to support the delivery of nonpandemic health care services in Europe during the COVID-19 pandemic. Specifically, it aimed to characterize modifications of care involving digital health, including the telehealth systems used and their functionalities, and how they were integrated into hybrid models of care. Additionally, it aimed to provide an overview of the maturity and extent of evaluations conducted on the implemented care modifications.

## Methods

### Study Design

A scoping review was conducted to examine reports on health delivery modifications incorporating digital health components, which were introduced due to the COVID-19 pandemic to deliver nonpandemic health care services. The review was conducted as part of the Horizon Europe RAPIDE (Regular and Unplanned Care Adaptive Dashboard for Cross-Border Emergencies) project and followed the PRISMA-ScR (Preferred Reporting Items for Systematic Reviews and Meta-Analyses Extension for Scoping Reviews) guidelines [17] (Checklist 1). The research protocol was registered on the Open Science Framework Registries [18].

### Eligibility Criteria

The main outcome of interest for included studies was modifications to health delivery involving a digital health intervention, implemented for the provision of nonpandemic care in the context of the COVID-19 pandemic in Europe. Evidence sources included peer-reviewed primary research studies. Aligned with this outcome and context, the following specific inclusion criteria were applied: (1) studies reporting primary data on the use of digital care modifications (ie, health care delivery modifications with digital health components), alongside descriptions of their implementation, intervention characteristics (eg, specific setting, patient groups, and clinical contexts), telehealth systems used, and their care functions; (2) studies describing digital care modifications introduced or scaled up in the context of the COVID-19 pandemic and aimed for nonpandemic care services; (3) studies focused on care for adult populations; and (4) studies reporting data from countries within the European Union, the European Economic Area, the Schengen area, or the United Kingdom.

In addition, the following exclusion criteria were considered: (1) non–peer-reviewed publications; (2) studies that did not separately report outcomes for eligible countries; (3) publications in a language other than English, Dutch, French, or Spanish; and (4) studies with a primary focus on mental health or pediatric health care. Studies with a focus on mental and pediatric care were excluded due to the distinct organization and service delivery models used in these types of care, as these studies would have introduced additional variability beyond the scope of this review.

### Information Sources

A comprehensive search was initially conducted on May 22, 2024, and later updated on January 14, 2026. The following electronic databases were searched, without applying multidatabase searching: PubMed or MEDLINE (Ovid), Embase (Ovid), CINAHL (EBSCOhost), Web of Science (Clarivate), and PsycINFO (Ovid). No other registries or online resources were consulted, and no additional data were sought through contacts. No additional information sources or search methods were used.

### Search

The search aimed to cover a broad range of literature, for which the concepts “delivery of health care,” “nonpandemic care,” and “COVID-19” were used to build the search strategy across all databases, including a combination of keywords and indexed vocabulary. No further limits, restrictions, or search filters were applied. No existing search strategies from other reviews were adapted or reused. The search was developed and reviewed by all authors in consultation with an information specialist, and no other external experts peer-reviewed the search. The original search strategy was reproduced for updating the search, which was consistent with the original search in all databases. The reproducible searches for all databases are available in Multimedia Appendix 1. Duplicates were removed by the researchers using EndNote (Clarivate) and Rayyan.ai (Qatar Computing Research Institute) deduplication features. All information related to the search used is reported in accordance to the PRISMA-S (Preferred Reporting Items for Systematic Reviews and Meta-Analyses Literature Search Extension) [19] (Checklist 2).

### Selection of Sources of Evidence

A broad search was conducted with the aim of capturing the diversity of service delivery modifications. First, all studies describing service delivery modifications (both digital and nondigital) and meeting all other eligibility criteria were preliminarily included. Next, studies were categorized according to the types of service delivery modifications, and those applying digital care modifications were selected for inclusion. This approach ensured the selection of studies that addressed digital health as part of broader service modification, even if it was not the primary focus.

As part of the screening, a pilot was first conducted for title and abstract screening to ensure clarity among researchers regarding eligibility criteria. Title and abstract screening was conducted by 3 researchers (NSV, TVL, and LN), and 10.4% (798/7652) of the total records were screened by at least two researchers independently. Full-text screening was conducted by 2 researchers (NSV and LN), with 5% (32/641) of the records screened by both researchers independently. The remaining records were screened separately due to strong agreement in the pilot screening. Citation searching was conducted for review studies that met the initial inclusion criteria through a manual search of reference lists; cited studies were then individually screened for inclusion. All discrepancies were resolved by consensus. The web application Rayyan.ai was used to support the screening process.

### Data Charting Process

A standardized data extraction form was developed and piloted among 3 researchers (NSV, TVL, and Dilek Yildirim), until agreement and consistency were reached. Studies were then split among the researchers who conducted the data extraction (NSV, TVL, and Dilek Yildirim). The online platform Airtable (Formagrid Inc) was used for data extraction, and Microsoft Excel was used to support additional data collation for reporting.

### Data Items

Data extracted included the year of publication, study design, country, health care discipline, level of care, pandemic period of study data, presence and type of evaluation, and characteristics of the digital care modifications (including telehealth systems, digital interventions, care functions, and in-person care components). Definitions for the uses of digital technology for health, including systems and interventions, were based on World Health Organization [3] and were used to inform both the terminology and categorization of digital components during data extraction and synthesis.

### Critical Appraisal of Individual Sources of Evidence

The methodological quality of the studies was appraised by 2 researchers (NSV and TVL) using the Mixed Methods Appraisal Tool (MMAT) [20]. The researchers independently rated a subset of 5 studies and aligned on the interpretation of criteria. The remaining studies were divided and separately appraised by the 2 researchers using a standardized form. Percentages of quality criteria met were calculated per study, and no studies were excluded based on methodological quality.

### Synthesis of Results

Descriptions of digital care modifications were inductively analyzed and used to create distinct *digital flows*, defined as the combination of 1 telehealth system, 1 digital intervention, and 1 care function (Table 1). Digital flows were constructed by the researchers based on descriptions of digital health modifications in each study. For studies describing several systems, interventions, or care functions, multiple digital flows were recorded. Digital care modifications were further categorized according to their hybrid care implementation, registering whether digital health was implemented as an individual approach (digital-only) or in combination with the provision of in-person care components (hybrid). Study evaluations of reported modifications were recorded following the Kirkpatrick model of evaluation, allowing multiple levels of evaluation, if applicable [21]. The Kirkpatrick model covers 4 distinct levels of evaluation and has been widely applied across health disciplines, allowing its pragmatic application across diverse outcomes as well as its straightforward interpretation [21].

**Table 1.** Definitions used to construct digital flows for mapping hybrid care modifications in the delivery of nonpandemic care.

Concept	Definition
Telehealth systems	Information and telecommunication technologies used to provide clinical care [3]
Digital health interventions	Technology capabilities designed to address a specific health objective [3]
Care function	Function or purpose of the digital health intervention: includes triage (prioritization of patients’ needs based on the urgency of their condition), first consultation (initial contact between a patient and a health care provider to evaluate a novel health concern), follow-up (subsequent care contacts to evaluate previously diagnosed conditions), among others

## Results

### Selection of Sources of Evidence

**Figure 1.**
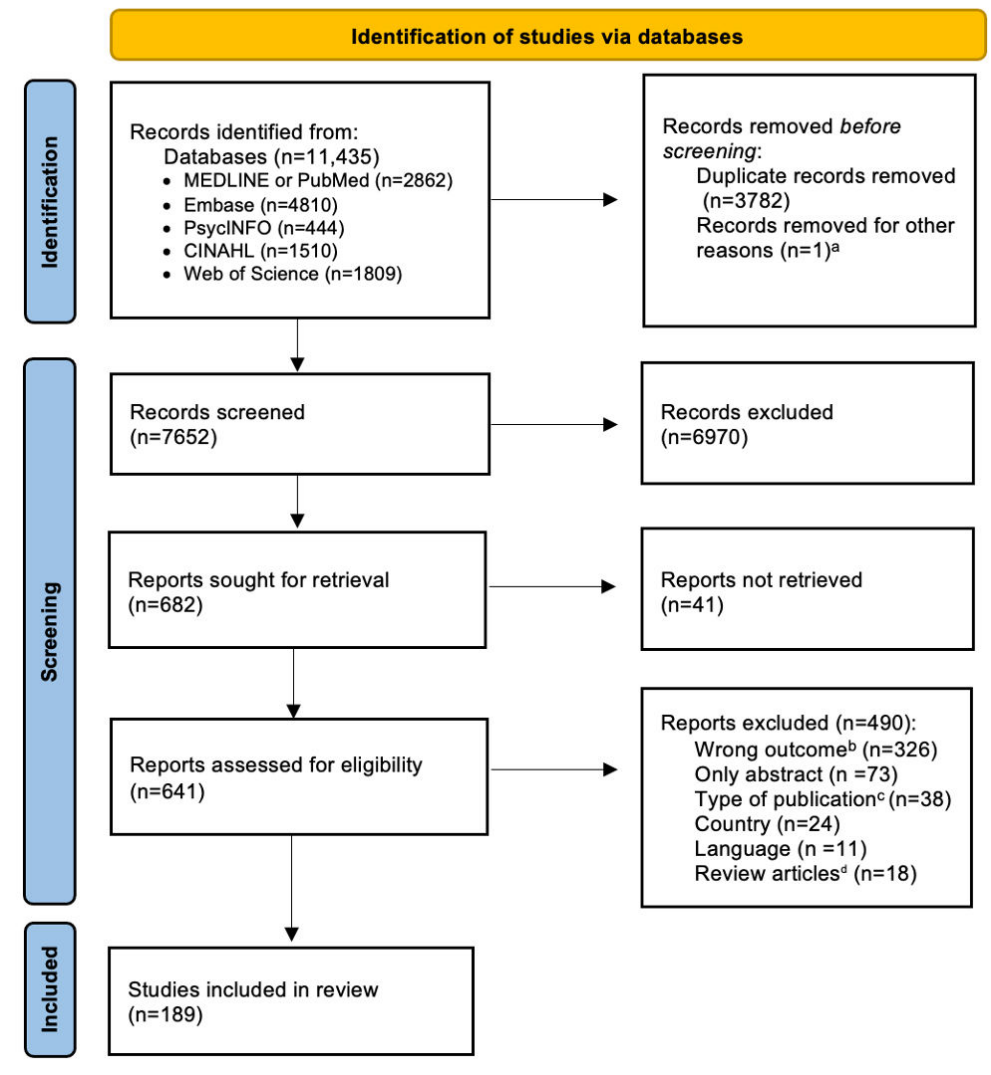
PRISMA (Preferred Reporting Items for Systematic Reviews and Meta-Analyses) flow diagram depicting the study selection process. ^a^Article retracted. ^b^Outcome defined as digital health modifications meeting inclusion criteria. ^c^Includes opinion pieces, editorials, letters to the editor, correspondence, commentaries, position papers, and other non–peer-reviewed publications. ^d^Review articles snowballed for additional records meeting inclusion criteria, with 38 additional records identified for inclusion.

In total, 7652 distinct studies were identified, of which 641 full texts were screened for eligibility. Studies included within identified reviews (n=18) were further screened for eligibility, resulting in 38 additional included studies. Finally, 189 studies met eligibility criteria and were included in the analysis (Figure 1).

### Characteristics of Sources of Evidence

Included studies were published between 2020 and 2025. Most studies were written in English (183/189, 96.9%), and several had full text available in Spanish (n=4) [22-25] and French (n=2) [26,27]. Over 80% (n=157) of the studies were either observational (n=79) or descriptive (n=78). The studies covered 23 distinct countries and 37 distinct health care disciplines across different settings, with a strong dominance of secondary and tertiary care (n=139) and nonsurgical disciplines (26/37, 70.3%). Data derived from the year 2020 were most frequently reported, and 114 studies exclusively covered data from February to June 2020. The studies’ characteristics are found in Table 2 (additional details provided in Multimedia Appendix 2).

**Table 2.** Characteristics of included studies in the scoping review.

Characteristics	Studies
Year of publication, n (%)	
2020	65 (34.4)
2021	57 (30.2)
2022	27 (14.3)
2023	17 (9)
2024	15 (7.9)
2025	8 (4.2)
Countries^a^, n	
United Kingdom	52
Italy	45
Spain	18
France	12
The Netherlands	8
Ireland	7
Portugal	6
Germany	5
Poland	5
Sweden	4
Austria	3
Belgium	3
Denmark	3
Norway	3
Romania	3
Switzerland	3
Bulgaria	1
Croatia	1
Czech Republic	1
Finland	1
Hungary	1
Malta	1
Slovenia	1
Studies focusing on 5 or more countries	6
Study design, n (%)	
Observational	79 (41.8)
Descriptive	78 (41.3)
Qualitative	14 (7.4)
Mixed methods	13 (6.9)
Experimental	5 (2.6)
Levels of care^b^, n	
Secondary or tertiary care	139
Primary care	57
Community care	5
Home care or residential care	1
Alternative medicine	1
Not specified	1
Health care disciplines^c^, n	
General practice or family medicine	46
Oncology	32
Cardiology	14
Neurology	14
Orthopedics and trauma	14
Endocrinology	8
Gastrointestinal medicine	8
Gynecology or obstetrics, including reproductive health	7
Dentistry or oral surgery	6
Dermatology	4
Midwifery	4
Ophthalmology	4
Plastic surgery and burn unit	4
Rheumatology	4
Urology	4
Immunology	3
Physiotherapy	3
Pharmacy services	3
Other^d^	26
Multiple (>5)	5
Not specified^e^	3
Pandemic period of reported data^f^, n	
February-June 2020	170
July-December 2020	59
January-June 2021	39
July-December 2021	26
January-December 2022	8
2023‐2024	8
Not specified	6

a For studies reporting more than 1 country (but <5), all countries were counted separately.

b 14 studies reported more than 1 level of care.

c For studies reporting more than 1 health care discipline (but <5), all disciplines were counted separately.

d Other health care disciplines, less frequently reported. Details provided in Multimedia Appendix 2.

e Specific areas of care not indicated.

f Refers to the period corresponding to the data reported in studies (not the date of publication). Studies may report data from several periods. The number of studies in each period is reported and therefore studies may be represented in different time periods.

### Critical Appraisal Within Sources of Evidence

Quality appraisal was conducted for 84.1% (159/189) of the studies, while 15.9% (30/189) comprised narrative accounts of care modifications and did not meet the MMAT screening criteria to be appraised. Nearly 60% (94/159) of the appraised studies met 80% or more of the quality criteria, and only a small number of studies (9/159) met 40% or less of the MMAT criteria.

### Synthesis of Results

#### Digital and Hybrid Care Modifications

Hybrid care modifications (incorporating both in-person and digital care components) were reported by 60.8% (115/189) of the studies, while digital-only care modifications (without in-person care components) were reported by 36% (68/189) of the studies. In 3.2% (6/189) of the studies, the extent of hybrid care use was not reported, and broad descriptions of digital care use did not allow classification into a specific category; although these studies could not be classified, they were still incorporated in the descriptive analysis of digital care modifications.

Care modifications incorporating hybrid modalities integrated digital and in-person components in a variety of ways. Hybrid care was used either for a determined subset of patients, for example, offering both in-person and digital care for the same patient but for different care purposes, or for different patient populations, for example, selecting either digital care or in-person care. Service delivery modifications also ranged from being predominantly focused on digital care while allowing in-person care in certain contexts [28-59] to approaches that incorporated a digital care component while predominantly maintaining in-person care [60-63]. For instance, several studies described the dominance of remote services, with in-person visits used only for specific clinical indications [28,36,48] or care purposes such as therapy administration [34] or clinical examination [41]. Most modifications, however, described the incorporation of substantial elements of both in-person and digital care.

Care modifications incorporating in-person and digital care components were commonly described in specialty care contexts. Organized care pathways, involving structured models for hybrid care provision, were found in areas, including orthopedic surgery [64,65], oncology [66,67], plastic surgery [68], and endocrinology [69]. Set clinical criteria, definition of patient groups, and applications of triage were common considerations reported to define the extent of application of hybrid care, both in primary and specialty care contexts.

Among modifications incorporating substantial in-person and digital care components, in-person care was commonly offered to patients requiring nondeferrable care [24,65,68,70-80], often decided after remote assessments [65,71,73,77]. Several studies reported in-person care only in clinically necessary cases [81-84]. Specific clinical criteria were often used to select in-person care contacts, including cases of severe disease or suspicion of malignancy [66,78,85-88]. Several studies focusing on cancer patients prioritized in-person consultations for new diagnoses or initial assessments [26,72,74,76,82]. Other patient factors were considered to select in-person versus remote care, including patient preference [82,87,89], patient age [69,90], and ease of access to health care facilities [91]. Conversely, remote consultations were more commonly reported for known patients [81,92] and scheduled follow-up consultations [83,93-99].

Studies commonly reported multiple in-person care components taking place within hybrid care provision, for either the same group of patients or for different patients within a health service. In general, in-person consultations and surgical interventions were the most frequently reported in-person care components involved in hybrid care use. In several cases, interventions such as physical examinations and functional tests were explicitly reported separately from overall consultations (Table 3).

**Table 3.** In-person care components reported in modifications involving hybrid care use for the delivery of nonpandemic care throughout the COVID-19 pandemic in Europe.

In-person care interventions	Studies, n
Consultation	85
Surgical intervention	23
Therapy administration	18
Minor procedures or interventions	17
Imaging	16
Laboratory tests	15
Physical examination	13
Inpatient interventions	7
Functional tests	5
Endoscopic procedures	3
Vital signs measurement	3
Not specified	6

**Figure 2.**
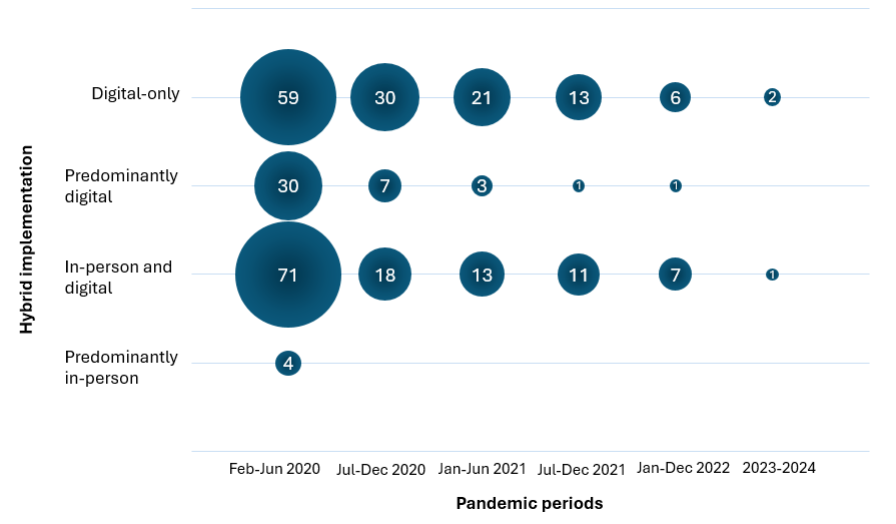
Variations in the report of hybrid care modifications for the delivery of nonpandemic care across different periods of the COVID-19 pandemic in Europe. Bubbles depict number of studies reporting different types of hybrid care modifications. Unspecified values are excluded.

In addition, hybrid and digital care modifications were implemented to varying degrees throughout the pandemic. Hybrid care (including in-person and digital, predominantly digital, and predominantly in-person modifications) was more frequently reported than digital-only care during the early stages of the pandemic (February to June 2020), while digital-only care was reported more often between July 2020 and June 2021. In 2022, hybrid care variations remained more common than digital-only modifications, although overall reporting decreased. Studies reporting care modifications in the postpandemic period (2023 and 2024) were scarce (Figure 2).

#### Digital Flows

**Figure 3.**
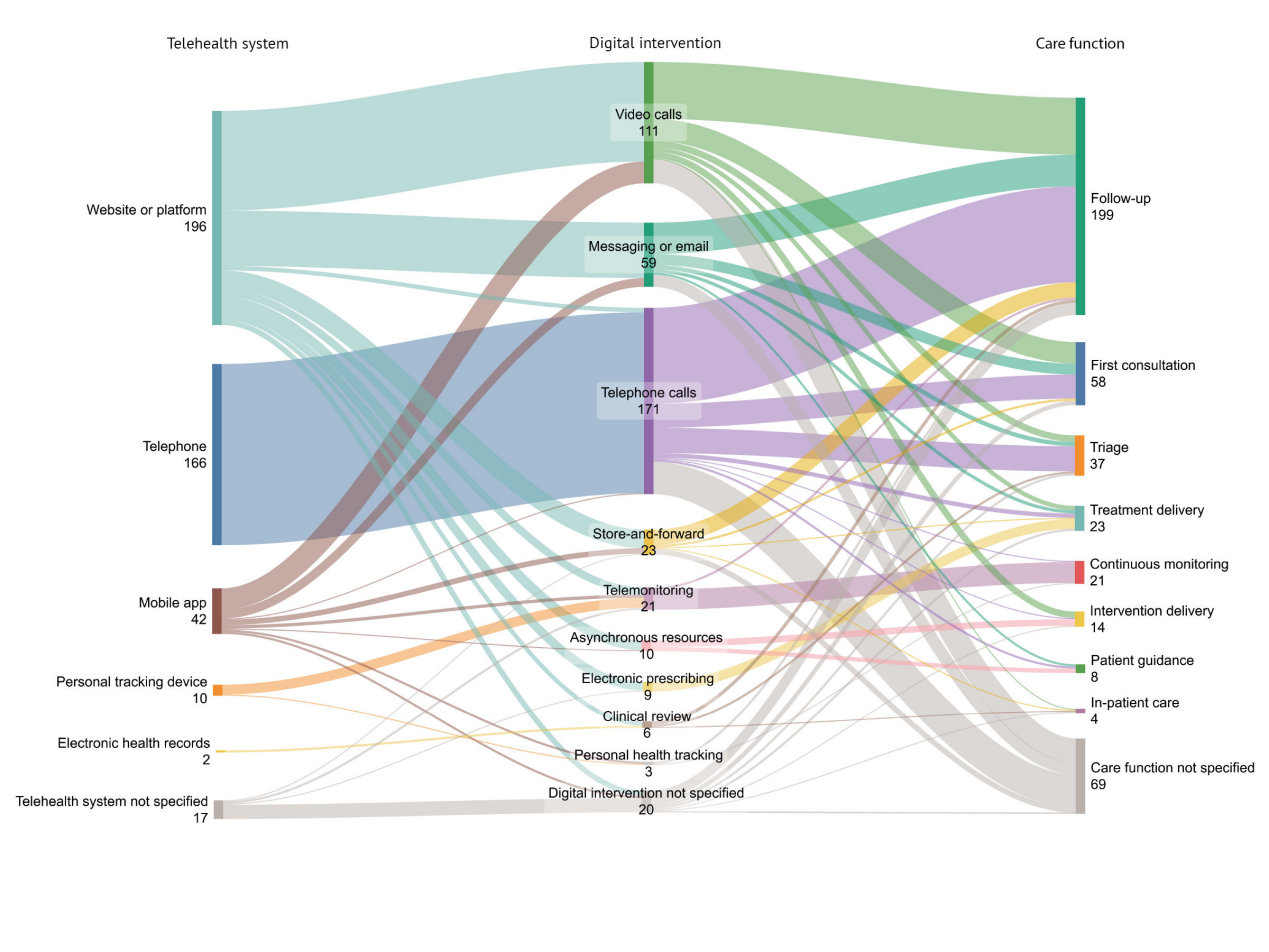
Digital flows illustrating digital care modifications implemented during the COVID-19 pandemic for the delivery of nonpandemic care in Europe. Digital flows are composed of 3 levels that indicate telehealth systems, digital interventions, and care functions. All telephone calls were assumed to have occurred via telephone service, if not indicated otherwise. Telehealth system for video calls was not indicated in 25 cases, for which the use of “website or platform” was assumed. Digital interventions were classified following the World Health Organization classification of digital interventions, services, and applications in health [3] whenever possible, with multiple interventions recorded when applicable.

Digital care modifications identified are illustrated through the creation of digital flows, composed of 3 levels that indicate telehealth systems, digital interventions, and care functions (Figure 3). A total of 433 individual digital care elements, comprising the 3 levels of telehealth systems, digital interventions, and care functions, were extracted, creating 68 distinct digital flows. Substantial variety was found in both interventions and care functions, while the diversity of telehealth systems was more limited. Distinct combinations of systems, interventions, and care functions formed several prominent flows.

One of the most prominent digital flows was the use of websites or platforms to conduct video calls, for the provision of follow-up care. This flow was commonly described in outpatient specialty care contexts, including neurology [22,23,29,100-102] and orthopedics [43,55,103,104]. Follow-up consultations in these cases often involved chronic patient care and postsurgical assessments, respectively. While specialty care was more common, video calls for follow-up purposes were also found at the primary and community care levels [51,95,105-111]. Additionally, the use of mobile technologies to support video calls was frequently linked to the follow-up care of patients in specialty contexts [35,43,49,82,94,100,103,112-114].

In addition to video calls, the role of messaging and email stood out. The application of these interventions was more evenly divided among primary and secondary or tertiary levels of care. At the primary care level, text-based interventions involved triage, first consultations, follow-up, and patient guidance as care functions. At the secondary or tertiary care level, interventions entailed follow-up of patients, including in cardiology [46,49,62,114-116] and oncology [32,34,117,118] services. Additionally, a number of text-based interventions highlighted mobile apps as the telehealth systems used [49,109,111,114,118-120].

Another key digital flow involved the use of telephone calls, commonly with the functions of follow-up, first consultations, and triage. The use of telephone calls was extensively reported at the secondary or tertiary care level across specialties including oncology [32,34,45,50,54,59,63,76,78,79,121-124], orthopedics [30,37,38,55,64,65,75,104,125-127], and neurology [22,29,53,100,101,128-132]. At the primary care level, telephone use often involved follow-up care, but first consultations and triage also took a substantial role.

Moreover, telemonitoring as a digital intervention was supported by personal tracking devices, websites or online platforms, and mobile apps and was most commonly used for continuous patient monitoring. Telemonitoring included live monitoring of patients’ clinical status [133], periodical monitoring to assess treatment adherence and adjust management plans [49], and a combination with other interventions such as telephone follow-up consultations [56,120]. In one instance, it also included the use of artificial intelligence (AI) for instant patient feedback [120].

#### Evaluation of Modifications

Over half of the studies (96/189, 50.8%) did not describe any kind of evaluation. Studies without evaluation were most commonly descriptive (n=55) and often exclusively considered data from February to June 2020 (n=64). Studies that evaluated modifications (93/189, 49.2%) were mostly limited to the year 2020 (n=66). Studies involving the use of certain telehealth systems more commonly reported evaluations, including 73.8% (31/42) of studies reporting the use of mobile apps and 61.4% (105/171) of studies reporting the use of websites or platforms.

**Figure 4.**
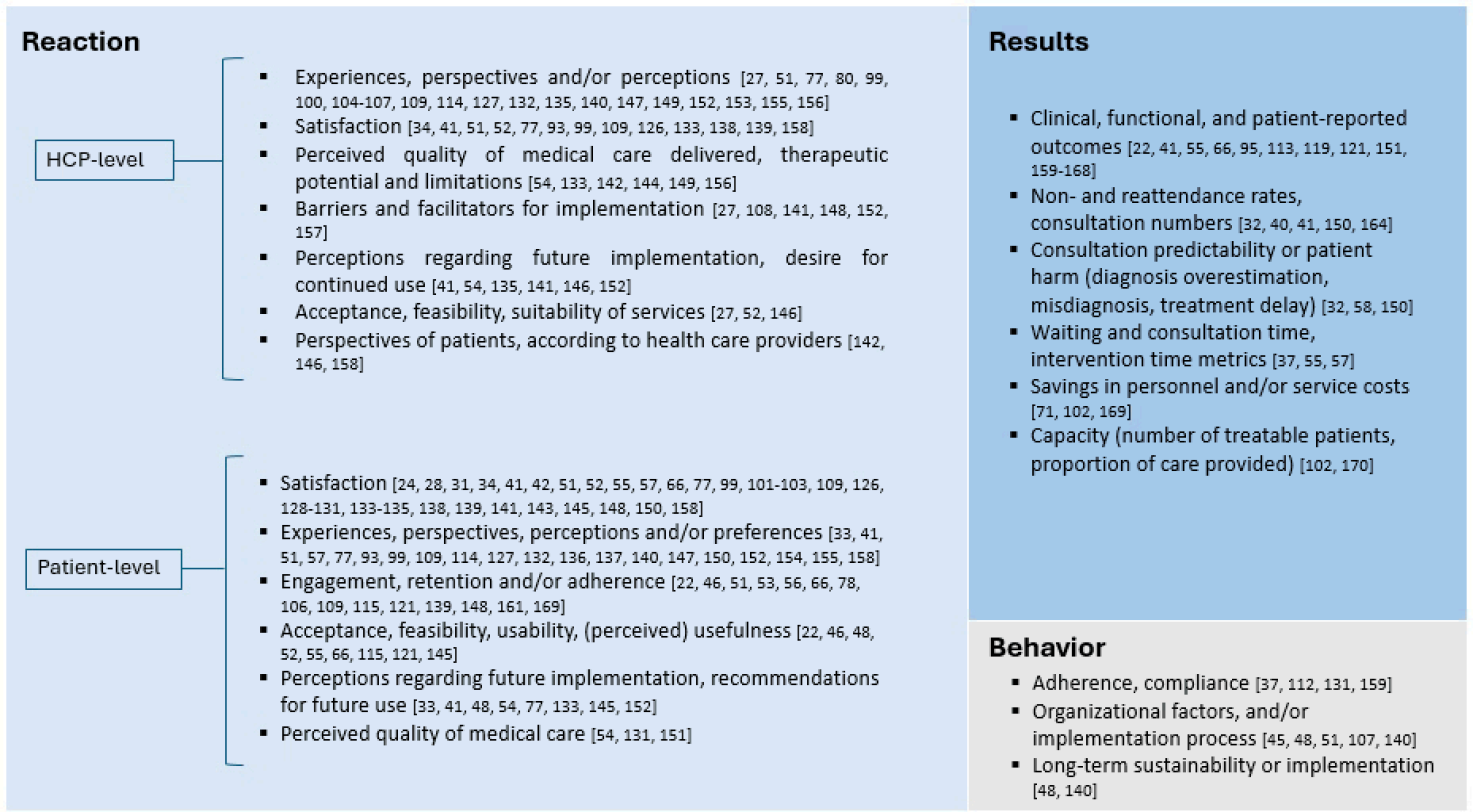
Levels of evaluation (Kirkpatrick model) and associated evaluated elements found in studies describing hybrid care modifications for the delivery of nonpandemic care during the COVID-19 pandemic in Europe. Each level size is represented according to the number of studies with evaluations at each level. A total of 18 studies reported multiple levels of evaluation. HCP: health care provider [23,25,28,31-34,37,40-42,45,46,48,51-58,66,67,71,77-80,93,95,99-109,112-115,119,121,126-171].

Among all studies with evaluations, evaluations at a reaction level (n=73) were the most common, followed by evaluations at the results level (n=29) and the behavior level (n=9; Figure 4). No studies included evaluations at a learning level. At the reaction level, methodologies for evaluation ranged from the use of surveys and questionnaires [23,25,28,29,32,34,35,42,43,47,49,52,53,55,56,58,67,78,94,100-104,110,127-154] to qualitative participant accounts, often collected through interviews and focus group discussions [49,52,67,81,100,105-110,119,122,133,142,149,153,155-159].

Studies evaluated at the reaction level commonly assessed satisfaction and experiences and perceptions of health care providers and patients. These studies generally reported high levels of satisfaction, as well as overall positive experiences. Concerning health care providers’ perspectives, some studies also reported disadvantages such as increased time investment and workload [42,81,156], increased cognitive demand [52], and concerns regarding contact and communication with patients [28,115,133]. From patients’ perspectives, studies highlighted benefits, including economic and time travel–related costs [78,115,130]. Additionally, some studies reported concerns related to limited uptake, impeded access, or lower satisfaction among specific patient groups, including the older population [25,35,43,131,134,136,159].

Multiple studies evaluated patients’ and providers’ perspectives on future implementation of digital health, in some cases proposing new hybrid models of care to be incorporated into new care standards [138]. Other studies used evaluations to build guidance for overcoming barriers for long-term implementation [49] and to join wider initiatives for adapting care services beyond the pandemic period [46]. Additionally, several studies explored the experiences of other stakeholders, including staff involved in the organization of care (eg, receptionists, practice managers, program managers, IT staff, medical advisors, health insurance representatives) [105,106,119,149,153,158] and caregivers [129,130].

In relation to evaluations at the results level, studies mainly focused on the measurement of clinical outcomes (including adverse events and laboratory measurements) [42,56,96,114,120,151,152,160-166], functional outcomes (including physical function measures) [23,67,122,167,168], and patient-reported outcomes (including quality of life and general health status) [42,56,67,122,169].

## Discussion

### Summary of Evidence

This scoping review has provided a comprehensive examination of modifications involving hybrid care implemented during the COVID-19 pandemic to support nonpandemic care services. To our knowledge, this is the first review focusing on hybrid care modifications implemented throughout the entire pandemic period. During the COVID-19 pandemic, digital health was considerably scaled up to support the provision of nonpandemic care, which could be observed through frequent reports of digital health as a novel or upscaled application. By mapping digital flows, we were able to examine the diverse ways digital care modifications were implemented in practice, highlighting the importance of analyzing the different components involved in digital health implementation. Linked to digital flows, the limited number of telehealth systems supporting a variety of care functions underscores their practical versatility. This review not only examines the variety of telehealth systems, interventions, and care functions used in different health care settings but also expands the understanding of their implementation as part of hybrid care pathways.

Our findings show that digital health was commonly described as part of hybrid care provision, rather than standalone digital approaches. Central factors for implementation included the selection of patient groups, prioritization based on clinical condition and urgency, and availability of telehealth systems, which often defined the distribution and use cases of in-person and digital interventions. The diverse ways in which in-person and digital care were combined reflect the constant need for adaptation of care models throughout the pandemic and across health care settings. Previous experiences with specific health care settings and patient groups may point to necessary context-specific adaptations [170-172]. Also linked to the constant need for adaptation, the number of publications describing hybrid care modifications decreased throughout the years. This decrease may be partly reflective of the return to prepandemic care delivery but may also be related to a decrease in the reporting of novel care delivery modifications.

The creation of digital flows revealed the various ways digital health technologies were implemented during the pandemic. Notably, a few telehealth systems facilitated the application of numerous interventions and care functions. Online websites and platforms were more commonly reported than mobile technologies, which may have been linked to unclear reporting of which specific systems were used and whether overlaps with other systems were present. Similarly, reports of mobile technology application may have been limited to specific demographic groups, such as the younger population. The concomitant use of multiple telehealth systems may reflect efforts to target distinct care functions and different types of users. Evidence on disparities of technology adoption for different users, including older people and ethnic minorities [173,174], underscores the need for flexibility, adaptation, and consideration of access requirements in the implementation of models of care during emergency situations.

Emerging technologies using AI, such as virtual health assistants, were nearly absent in our findings. Previous studies indicate the use of these technologies during the pandemic was concentrated in areas other than direct nonpandemic care, such as diagnosis and detection of COVID-19 [175], which may also be related to their early stage of development, as well as regulatory barriers hindering wider adoption. As technology advances, it is expected that these types of interventions might take on a more prominent role in the future.

In contrast to telehealth systems, a wider variety of digital interventions was identified. The frequent use of telephones is in line with findings from previous studies [176,177] and may be associated with their familiarity, low cost, and ease of access for providers and patients. The common report of the use of video calls during the pandemic has also been previously reported in multiple health care contexts [177-179]. While text-based interventions have been examined in previous studies [176], more research may be needed to better understand their scope, especially in relation to other interventions such as those involving store-and-forward technologies.

Interventions were most commonly linked to follow-up care, aligning with studies from the first months of the pandemic [176,177,180]. Previous research has pointed out that remote care may yield more positive outcomes when patients have a pre-existing relationship with providers [181]. Distinguishing between new patients and follow-up or known patients may also be of value for assessing satisfaction with remote consultations [55,131]. The broad application of different interventions for follow-up care indicates great potential for enabling continuity of care for patients with chronic conditions, both during normal and crisis situations.

Additionally, our findings show that evaluations of modifications in the delivery of nonpandemic care were limited in scope. Over half of the included studies did not report any form of evaluation, and most of those that did focus on short-term user experiences rather than behavioral, clinical, or long-term implementation outcomes. Many of these care modifications were introduced rapidly as emergency responses to an evolving crisis, often without the time or infrastructure to plan for formal evaluation. Time pressure and resource scarcity during the pandemic, alongside the frequent ad hoc nature of modifications, may have contributed to the low number of evaluations.

Evaluations of patients’ and providers’ experiences and satisfaction align with previous studies, which have similarly centered on user acceptability and adoption [182] and have found high levels of satisfaction with digital health during the pandemic [183,184]. Behavioral and organizational outcomes also align with evaluations conducted before the pandemic, which have evaluated aspects such as behavior change, cost-effectiveness, and clinical and patient-centered outcomes [185]. Regarding clinical outcomes, previous evidence has shown clinical efficacy in the use of digital health interventions for a variety of patient groups [186,187], highlighting its relevance for remote care provision. Although less frequently found, evaluations conducted with the aim of exploring long-term implementation underscore the critical need for addressing sustainability in digital health implementation [188].

### Limitations

This review has applied a comprehensive and systematic methodological approach. However, some limitations are worth noting. Although the inclusion of solely peer-reviewed publications helped ensure rigor in the synthesized data, evidence reported through other means has not been included. Additionally, a publication bias may exist toward positive experiences with digital health, and less successful practices may be underrepresented. The variation in study designs, alongside the diversity of health care contexts and patient groups, may have influenced the interpretation of the findings. Future research may provide additional insights into which specific patient groups may benefit more from the implementation of hybrid care.

This review limited its scope to modifications directly related to service provision; however, other types of interventions, such as those for interprofessional collaboration or patient education, may also be valuable for improving timely access to high-quality care. Strategies to improve remote communication between primary and secondary care providers, as well as the implementation of remote multidisciplinary meetings, have shown potential to improve the organization of care and may be a focus for future research [34,42,75,80,99,121].

Moreover, differences in the use of terminology for digital health greatly contribute to diverse reporting of systems, interventions, and functions and may have influenced the reporting of results. Future research can benefit from using common terminology frameworks, increasing consistency in reporting and helping improve future understanding of the implementation and evaluation of digital and hybrid care. Such common frameworks may also be essential for the development of legal and regulatory procedures [180].

### Conclusions

This review synthesizes findings essential for understanding the scope of hybrid care modifications used in the delivery of nonpandemic care and implemented throughout the COVID-19 pandemic. It provides a unique view as it analyzes digital health interventions as part of broader care pathways, which combine different types of care delivery implemented to sustain access to care. Integrating digital health as part of hybrid care models is essential in designing care pathways that can adapt to different contexts, including future health emergency situations.

The use of digital health and emerging technologies such as AI will continue to shape the future of health care delivery. As evidence regarding its applications advances, research should identify which combinations of digital interventions and care functions offer the greatest accessibility and cost-effectiveness. In the future, research may expand the application of hybrid care models to innovative strategies for the effective management of any crisis involving disrupted infrastructure. Policymakers, researchers, and implementers should also prioritize strategies to reduce inequities in access to digital and in-person care, particularly for underserved populations. Finally, evaluations should move beyond short-term user experiences and assess long-term implementation, clinical outcomes, and sustainability of hybrid care models.

## Supplementary material

10.2196/84756 Multimedia Appendix 1 Search strategy.

10.2196/84756 Multimedia Appendix 2 Extracted data of included studies.

10.2196/84756 Checklist 1 PRISMA-ScR checklist.

10.2196/84756 Checklist 2 PRISMA-S checklist.

## Abbreviations

AI: artificial intelligence
MMAT: Mixed Methods Appraisal Tool
PRISMA-S: Preferred Reporting Items for Systematic Reviews and Meta-Analyses Literature Search Extension
PRISMA-ScR: Preferred Reporting Items for Systematic Reviews and Meta-Analyses Extension for Scoping Reviews
RAPIDE: Regular and Unplanned Care Adaptive Dashboard for Cross-Border Emergencies
