# Correction: Hybrid Care Modifications in the Delivery of Nonpandemic Care During the COVID-19 Pandemic: Scoping Review

**DOI:** 10.2196/101728

**Published:** 2026-06-04

**Authors:** Natalia Sanchez Villalobos, Tessa van Loenen, Lem Ngongalah, Máire A Connolly, Mart L Stein, Chantal P Rovers, Aura Timen

**Affiliations:** 1 Department of Primary and Community Care, Radboud University Medical Center, Geert Grooteplein Noord 21, Nijmegen, 6525EZ, The Netherlands, 31 0243618181; 2 School of Health Sciences, University of Galway, Galway, Ireland; 3 Centre for Infectious Disease Control, National Institute for Public Health and the Environment, Bilthoven, The Netherlands; 4 Department of Internal Medicine, Radboud University Medical Center, Nijmegen, The Netherlands

In “Hybrid Care Modifications in the Delivery of Nonpandemic Care During the COVID-19 Pandemic: Scoping Review” [1], the authors noted one error.

The funding information has been added to the end of the article as follows:

*This study was funded by the European Union. Views and opinions expressed are, however, those of the authors only and do not necessarily reflect those of the European Union or the European Health and Digital Executive Agency (HaDEA). Neither the European Union nor the granting authority can be held responsible for them. The funder had no involvement in the study design, data collection, analysis, interpretation, or writing of the manuscript.*

The correction will appear in the online version of the paper on the JMIR Publications website, together with the publication of this correction notice. Because this was made after submission to PubMed, PubMed Central, and other full-text repositories, the corrected article has also been resubmitted to those repositories.

## References

[1] Sanchez Villalobos N, van Loenen T, Ngongalah L (2026). Hybrid care modifications in the delivery of nonpandemic care during the COVID-19 pandemic: scoping review. J Med Internet Res.

