# Supplementary material for: Hybrid Care Modifications in the Delivery of Nonpandemic Care During the COVID-19 Pandemic: Scoping Review
Source: J Med Internet Res. 2026 Apr 30;28:e84756. doi: 10.2196/84756 (PMC13131888; doi:10.2196/84756)
Supplement: Multimedia Appendix 1 [file jmir-v28-e84756-s001.doc]

**Multimedia appendix**

**Search strategy**

**Table S1.** Search strategy MEDLINE

| **Key concepts** | **Search string** |
| --- | --- |
| Delivery of healthcare | (Health* delivery or health* services or care services or health* provision or service provision or health consultation* or health appointment* or Delivery of health* or delivery of care or telehealth or telemedicine or mhealth or m-health or mobile health or ehealth or e-health or electronic health or digital health).ti,ab,kf. or "exp delivery of health care"/ or health services/ or adult day care centers/ or exp community health nursing/ or exp patient participation/ or community pharmacy services/ or home care services/ or home nursing/ or exp maternal health services/ or exp dental health services/ or emergency medical services/ or emergency medical dispatch/ or emergency room visits/ or emergency service, hospital/ or triage/ or health services for persons with disabilities/ or health services for the aged/ or health services for transgender persons/ or military health services/ or exp nursing care/ or exp nursing services/ or patient care/ or ambulatory care/ or exp "continuity of patient care"/ or day care, medical/ or delayed diagnosis/ or "duration of therapy"/ or "episode of care"/ or foster home care/ or hospitalization/ or long-term care/ or palliative care/ or exp perinatal care/ or exp perioperative care/ or prenatal care/ or subacute care/ or terminal care/ or time-to-treatment/ or pharmaceutical services/ or prescriptions/ or preventive health services/ or exp diagnostic services/ or mass screening/ or mobile health units/ or early medical intervention/ or health services for prisoners/ or rehabilitation/ or exp reproductive health services/ or exp rural health services/ or urban health services/ or veterans health services/ or women's health services/ |
| AND | |
| Non-pandemic care | (((Regular or routine or usual) adj3 (care or healthcare or services or check up* or appointment* or consult*)) or (urgent care or semi-urgent care or non-urgent care or preventive care or standard care or continuity of care or standard treatment or traditional healthcare or traditional care or chronic care or chronic condition* or chronic disease* or chronic treatment or chronic illness* or chronically ill or chronic patient* or emergency care or ongoing care or health maintenance or health monitoring or health screening or patient monitoring or primary care or primary health* or medic* prescription* or essential care or essential health* or essential service* or necessary health service* or outpatient care or inpatient care or ambulatory care or noncommunicable disease* or non-communicable disease* or non-COVID-19 or non COVID-19 or long-term care or long term care or home care or in-home care)).ti,ab,kf. or Chronic Disease/ or Noncommunicable Diseases/ or ambulatory care/ or "continuity of patient care"/ or Mass Screening/ or Primary Health Care/ |
| AND | |
| COVID-19 | COVID-19/ or ((coronavirus/ or betacoronavirus/ or coronavirus infections/) and (disease outbreaks/ or epidemics/ or pandemics/)) or ((coronavirus* or corona virus* or betacoronavirus*) adj3 (pandemic* or epidemic* or outbreak* or crisis)).ti,ab,kf. |
| AND | |
| Europe | (Europe* or EU or EEA or Schengen or UK or Austria* OR Belgium OR Belgian* OR Bulgaria* OR Croatia* OR Cyprus OR Cypriot* OR Czech* OR Denmark OR Danish OR Estonia* OR Finland OR Finnish OR France OR French OR German* OR Greece OR Greek* OR Hungary OR Hungarian* OR Iceland* OR Ireland OR Irish OR Italy OR Italian* OR Latvia* OR Liechtenstein* OR Lithuania* OR Luxembourg* OR Malta OR Maltese OR Netherlands OR Dutch OR Norway OR Norwegian* OR Poland OR Polish OR Portugal OR Portuguese OR Romania* OR Slovakia OR Slovak* OR Slovenia* OR Spain OR Spanish OR Sweden OR Swedish OR Switzerland OR Swiss OR United Kingdom OR British OR Great Britain OR England OR English OR Scotland OR Scottish OR Wales OR Welsh).ti,ab,kf. or exp Europe/ |

**Table S2**. Search strategy EMBASE

| **Key concepts** | **Search string** |
| --- | --- |
| Delivery of healthcare | (Health* delivery or health* services or care services or health* provision or service provision or health consultation* or health appointment* or Delivery of health* or delivery of care or telehealth or telemedicine or mhealth or m-health or mobile health or ehealth or e-health or electronic health or digital health).ti,ab,kf. or Health care delivery/ or exp aftercare/ or age specific care/ or exp ambulatory care/ or care bundle/ or day care/ or hospital care/ or exp nursing care/ or out-of-hours care/ or patient triage/ or practice gap/ or residential care/ or surge capacity/ or telehealth/ or telemedicine/ or community care/ or community based rehabilitation/ or community based surveillance/ or community health nursing/ or home care/ or home monitoring/ or home rehabilitation/ or home visit/ or health service/ or clinical pharmacy/ or dietary service/ or emergency health service/ or family service/ or hospital service/ or maternal health service/ or medical service/ or exp military health service/ or health care utilization/ or patient care/ |
| AND | |
| Non-pandemic care | (((Regular or routine or usual) adj3 (care or healthcare or services or check up* or appointment* or consult*)) or (urgent care or semi-urgent care or non-urgent care or preventive care or standard care or continuity of care or standard treatment or traditional healthcare or traditional care or chronic care or chronic condition* or chronic disease* or chronic treatment or chronic illness* or chronically ill or chronic patient* or emergency care or ongoing care or health maintenance or health monitoring or health screening or patient monitoring or primary care or primary health* or medic* prescription* or essential care or essential health* or essential service* or necessary health service* or outpatient care or inpatient care or ambulatory care or noncommunicable disease* or non-communicable disease* or non-COVID-19 or non COVID-19 or long-term care or long term care or home care or in-home care)).ti,ab,kf. or chronic disease/ or non communicable disease/ or exp ambulatory care/ or outpatient care/ or mass screening/ or exp primary health care/ |
| AND | |
| COVID-19 | coronavirus disease 2019/ or ((coronavirinae/) and (pandemic/ or epidemic/)) or ((coronavirus* or corona virus* or betacoronavirus*) adj3 (pandemic* or epidemic* or outbreak* or crisis)).ti,ab,kf. |
| AND | |
| Europe | (Europe* or EU or EEA or Schengen or UK or Austria* OR Belgium OR Belgian* OR Bulgaria* OR Croatia* OR Cyprus OR Cypriot* OR Czech* OR Denmark OR Danish OR Estonia* OR Finland OR Finnish OR France OR French OR German* OR Greece OR Greek* OR Hungary OR Hungarian* OR Iceland* OR Ireland OR Irish OR Italy OR Italian* OR Latvia* OR Liechtenstein* OR Lithuania* OR Luxembourg* OR Malta OR Maltese OR Netherlands OR Dutch OR Norway OR Norwegian* OR Poland OR Polish OR Portugal OR Portuguese OR Romania* OR Slovakia OR Slovak* OR Slovenia* OR Spain OR Spanish OR Sweden OR Swedish OR Switzerland OR Swiss OR United Kingdom OR British OR Great Britain OR England OR English OR Scotland OR Scottish OR Wales OR Welsh).ti,ab,kf. or exp Europe/ |

**Table S3.** Search strategy PsychINFO

| **Key concepts** | **Search string** |
| --- | --- |
| Delivery of healthcare | (Health* delivery or health* services or care services or health* provision or service provision or health consultation* or health appointment* or Delivery of health* or delivery of care or telehealth or telemedicine or mhealth or m-health or mobile health or ehealth or e-health or electronic health or digital health).ti,ab,id. or exp Health Care Delivery/ or exp health care services/ or community services/ or emergency services/ or home care/ or home visiting programs/ or hospital admission/ or hospital discharge/ or rural health/ or urban health/ or outpatient treatment/ or preventive health services/ or exp health screening/ or prenatal care/ or "prescribing (drugs)"/ or disease management/ or medical diagnosis/ or exp screening/ or exp electronic health services/ or exp aftercare/ or caregiving/ |
| AND | |
| Non-pandemic care | (((Regular or routine or usual) adj3 (care or healthcare or services or check up* or appointment* or consult*)) or (urgent care or semi-urgent care or non-urgent care or preventive care or standard care or continuity of care or standard treatment or traditional healthcare or traditional care or chronic care or chronic condition* or chronic disease* or chronic treatment or chronic illness* or chronically ill or chronic patient* or emergency care or ongoing care or health maintenance or health monitoring or health screening or patient monitoring or primary care or primary health* or medic* prescription* or essential care or essential health* or essential service* or necessary health service* or outpatient care or inpatient care or ambulatory care or noncommunicable disease* or non-communicable disease* or non-COVID-19 or non COVID-19 or long-term care or long term care or home care or in-home care)).ti,ab,id. or chronic illness/ or "continuum of care"/ or outpatient treatment/ or exp disease screening/ or primary health care/ |
| AND | |
| COVID-19 | COVID-19/ or ((coronavirus/) and (disease outbreaks/ or epidemics/ or pandemics/)) or ((coronavirus* or corona virus* or betacoronavirus*) adj3 (pandemic* or epidemic* or outbreak* or crisis)).ti,ab,id. |
| AND | |
| Europe | (Europe* or EU or EEA or Schengen or UK or Austria* OR Belgium OR Belgian* OR Bulgaria* OR Croatia* OR Cyprus OR Cypriot* OR Czech* OR Denmark OR Danish OR Estonia* OR Finland OR Finnish OR France OR French OR German* OR Greece OR Greek* OR Hungary OR Hungarian* OR Iceland* OR Ireland OR Irish OR Italy OR Italian* OR Latvia* OR Liechtenstein* OR Lithuania* OR Luxembourg* OR Malta OR Maltese OR Netherlands OR Dutch OR Norway OR Norwegian* OR Poland OR Polish OR Portugal OR Portuguese OR Romania* OR Slovakia OR Slovak* OR Slovenia* OR Spain OR Spanish OR Sweden OR Swedish OR Switzerland OR Swiss OR United Kingdom OR British OR Great Britain OR England OR English OR Scotland OR Scottish OR Wales OR Welsh).ti,ab,id. or european cultural groups/ |

**Table S4. Search strategy CINAHL**

| **Key concepts** | **Search string** |
| --- | --- |
| Delivery of healthcare | (TI ("Health* delivery" OR "health* services" OR "care services" OR "health* provision" OR "service provision" OR "health consultation*" OR "health appointment*" OR "Delivery of health*" OR "delivery of care" OR telehealth OR telemedicine OR mhealth OR "m-health" OR "mobile health" OR ehealth OR "e-health" OR "electronic health" OR "digital health")) OR  (AB ("Health* delivery" OR "health* services" OR "care services" OR "health* provision" OR "service provision" OR "health consultation*" OR "health appointment*" OR "Delivery of health*" OR "delivery of care" OR telehealth OR telemedicine OR mhealth OR "m-health" OR "mobile health" OR ehealth OR "e-health" OR "electronic health" OR "digital health")) OR  (MH "Health Care Delivery") OR (MH "Health Care Delivery, Integrated") OR (MH "Health Resource Utilization+") OR (MH "Health Services Accessibility+") OR (MH "Managed Care Programs") OR (MH "National Health Programs") OR (MH "Primary Health Care") OR (MH "Secondary Health Care") OR (MH "Telehealth") OR (MH "Telemedicine+") OR (MH "Telenursing+") OR (MH "Tertiary Health Care") OR (MH "Office Visits") OR (MH "Shared Medical Appointments") OR (MH "Appointments and Schedules") OR (MH "Patient Care") OR (MH "Age Specific Care") OR (MH "Case Management") OR (MH "Childbirth") OR (MH "Continuity of Patient Care+") OR (MH "Disease Management+") OR (MH "Emergency Room Visits") OR (MH "Family Centered Care") OR (MH "Gender Specific Care") OR (MH "Life Support Care") OR (MH "Multidisciplinary Care Team+") OR (MH "Nursing Care") OR (MH "Nursing Care Delivery Systems+") OR (MH "Gender Affirming Care") OR (MH "Patient Centered Care") OR (MH "Patient Classification") OR (MH "Patient Navigation") OR (MH "Patient Care Plans") OR (MH "Practice Guidelines") OR (MH "Health Services") OR (MH "Community Health Services+") OR (MH "Dental Health Services") OR (MH "Emergency Medical Services+") OR (MH "Health Services for the Indigent") OR (MH "Health Services for LGBTQ+ Persons") OR (MH "Health Services for Older Persons") OR (MH "Health Services for Persons with Disabilities") OR (MH "Hospital Programs") OR (MH "Institutionalization+") OR (MH "Military Health Services") OR (MH "Nutrition Services") OR (MH "Office Visits") OR (MH "Rehabilitation") OR (MH "Rural Health Services") OR (MH "Sexual Health Services") OR (MH "Urban Health Services") OR (MH "Veterans Health Services") OR (MH "Women's Health Services") OR (MH "Health Screening+") OR (MH "Diagnostic Services+") OR (MH "Prescriptions, Drug") OR (MH "Acute Care") OR (MH "After Care") OR (MH "Day Care") OR (MH "Foster Home Care") OR (MH "Long Term Care") OR (MH "Maternal-Child Care") OR (MH "Medical Care+") OR (MH "Terminal Care+") |
| AND | |
| Non-pandemic care | (TI ((Regular OR routine OR usual) N3 (care OR healthcare OR services OR check up* OR appointment* OR consult*))) OR (AB ((Regular OR routine OR usual) N3 (care OR healthcare OR services OR check up* OR appointment* OR consult*))) OR  (TI (“urgent care” OR "semi-urgent care" OR "non-urgent care” OR "preventive care" OR "standard care" OR "continuity of care" OR "standard treatment" OR "traditional healthcare" OR "traditional care" OR "chronic care" OR "chronic condition*" OR "chronic disease*" OR "chronic treatment" OR "chronic illness*" OR "chronically ill" OR "chronic patient*" OR "emergency care" OR "ongoing care" OR "health maintenance" OR "health monitoring" OR "health screening" OR "patient monitoring" OR "primary care" OR "primary health*" OR “medic* prescription*”OR "essential care" OR "essential health*" OR "essential service*" OR "necessary health service*" OR "outpatient care" OR "inpatient care" OR "ambulatory care" OR "noncommunicable disease*" OR "non-communicable disease*" OR "non-COVID-19" OR "non COVID-19" OR "long-term care" OR "long term care" OR "home care" OR "in-home care")) OR  (AB (“urgent care” OR "semi-urgent care" OR "non-urgent care” OR "preventive care" OR "standard care" OR "continuity of care" OR "standard treatment" OR "traditional healthcare" OR "traditional care" OR "chronic care" OR "chronic condition*" OR "chronic disease*" OR "chronic treatment" OR "chronic illness*" OR "chronically ill" OR "chronic patient*" OR "emergency care" OR "ongoing care" OR "health maintenance" OR "health monitoring" OR "health screening" OR "patient monitoring" OR "primary care" OR "primary health*" OR “medic* prescription*”OR "essential care" OR "essential health*" OR "essential service*" OR "necessary health service*" OR "outpatient care" OR "inpatient care" OR "ambulatory care" OR "noncommunicable disease*" OR "non-communicable disease*" OR "non-COVID-19" OR "non COVID-19" OR "long-term care" OR "long term care" OR "home care" OR "in-home care")) OR  (MH "Noncommunicable Diseases") OR (MH "Chronic Disease") OR (MH "Ambulatory Care") OR (MH "Health Screening+") OR (MH "Continuity of Patient Care") OR (MH "Primary Health Care") |
| AND | |
| COVID-19 | (TI ((coronavirus* or corona virus* or betacoronavirus*) N3 (pandemic* or epidemic* or outbreak* or crisis))) OR (AB ((coronavirus* or corona virus* or betacoronavirus*) N3 (pandemic* or epidemic* or outbreak* or crisis))) OR (MH "COVID-19") OR (MH "COVID-19 Pandemic") OR ((MH "Coronavirus Infections") AND (MH "Disease Outbreaks")) |
| AND | |
| Europe | (TI (Europe* OR EU OR EEA OR Schengen OR UK OR Austria* OR Belgium OR Belgian* OR Bulgaria* OR Croatia* OR Cyprus OR Cypriot* OR Czech* OR Denmark OR Danish OR Estonia* OR Finland OR Finnish OR France OR French OR German* OR Greece OR Greek* OR Hungary OR Hungarian* OR Iceland* OR Ireland OR Irish OR Italy OR Italian* OR Latvia* OR Liechtenstein* OR Lithuania* OR Luxembourg* OR Malta OR Maltese OR Netherlands OR Dutch OR Norway OR Norwegian* OR Poland OR Polish OR Portugal OR Portuguese OR Romania* OR Slovakia OR Slovak* OR Slovenia* OR Spain OR Spanish OR Sweden OR Swedish OR Switzerland OR Swiss OR United Kingdom OR British OR Great Britain OR England OR English OR Scotland OR Scottish OR Wales OR Welsh)) OR  (AB (Europe* OR EU OR EEA OR Schengen OR UK OR Austria* OR Belgium OR Belgian* OR Bulgaria* OR Croatia* OR Cyprus OR Cypriot* OR Czech* OR Denmark OR Danish OR Estonia* OR Finland OR Finnish OR France OR French OR German* OR Greece OR Greek* OR Hungary OR Hungarian* OR Iceland* OR Ireland OR Irish OR Italy OR Italian* OR Latvia* OR Liechtenstein* OR Lithuania* OR Luxembourg* OR Malta OR Maltese OR Netherlands OR Dutch OR Norway OR Norwegian* OR Poland OR Polish OR Portugal OR Portuguese OR Romania* OR Slovakia OR Slovak* OR Slovenia* OR Spain OR Spanish OR Sweden OR Swedish OR Switzerland OR Swiss OR United Kingdom OR British OR Great Britain OR England OR English OR Scotland OR Scottish OR Wales OR Welsh)) OR (MH "Europe+") |

**Table S5.** Search strategy Web of Science

| **Key concepts** | **Search string** |
| --- | --- |
| Delivery of healthcare | TS=("Health* delivery" OR "health* services" OR "care services" OR "health* provision" OR "service provision" OR "health consultation*" OR "health appointment*" OR "Delivery of health*" OR "delivery of care" OR telehealth OR telemedicine OR mhealth OR "m-health" OR "mobile health" OR ehealth OR "e-health" OR "electronic health" OR "digital health") |
| AND | |
| Non-pandemic care | TS=(((Regular OR routine OR usual) NEAR/2 (care OR healthcare OR services OR "check up" OR appointment OR consult)) OR (“urgent care” OR "semi-urgent care" OR "non-urgent care” OR "preventive care" OR "standard care" OR "continuity of care" OR "standard treatment" OR "traditional healthcare" OR "traditional care" OR "chronic care" OR "chronic condition*" OR "chronic disease*" OR "chronic treatment" OR "chronic illness*" OR "chronically ill" OR "chronic patient*" OR "emergency care" OR "ongoing care" OR "health maintenance" OR "health monitoring" OR "health screening" OR "patient monitoring" OR "primary care" OR "primary health*" OR “medic* prescription*”OR "essential care" OR "essential health*" OR "essential service*" OR "necessary health service*" OR "outpatient care" OR "inpatient care" OR "ambulatory care" OR "noncommunicable disease*" OR "non-communicable disease*" OR "non-COVID-19" OR "non COVID-19" OR "long-term care" OR "long term care" OR "home care" OR "in-home care")) |
| AND | |
| COVID-19 | **TS=(((coronavirus* OR "corona virus*" OR betacoronavirus*) NEAR/2 (pandemic* OR epidemic* OR outbreak* OR crisis)) OR (COVID-19 OR “coronavirus disease 2019” OR “coronavirus 2019”))** |
| AND | |
| Europe | TS=((Europe* OR "European Union" OR EU OR EEA OR Schengen OR UK OR Austria* OR Belgium OR Belgian* OR Bulgaria* OR Croatia* OR Cyprus OR Cypriot* OR Czech* OR Denmark OR Danish OR Estonia* OR Finland OR Finnish OR France OR French OR German* OR Greece OR Greek* OR Hungary OR Hungarian* OR Iceland* OR Ireland OR Irish OR Italy OR Italian* OR Latvia* OR Liechtenstein* OR Lithuania* OR Luxembourg* OR Malta OR Maltese OR Netherlands OR Dutch OR Norway OR Norwegian* OR Poland OR Polish OR Portugal OR Portuguese OR Romania* OR Slovakia OR Slovak* OR Slovenia* OR Spain OR Spanish OR Sweden OR Swedish OR Switzerland OR Swiss OR "United Kingdom" OR British OR "Great Britain" OR England OR English OR Scotland OR Scottish OR Wales OR Welsh) ) |
