# Supplementary material for: Hybrid Care Modifications in the Delivery of Nonpandemic Care During the COVID-19 Pandemic: Scoping Review
Source: J Med Internet Res. 2026 Apr 30;28:e84756. doi: 10.2196/84756 (PMC13131888; doi:10.2196/84756)
Supplement: Multimedia Appendix 2 [file jmir-v28-e84756-s002.docx]

**Multimedia appendix**

**Extracted data of included studies**

**Table S6.** General data extracted for included studies

| **Reference number** | **First author, year** | **Country(ies)** | **Study design** | **Level of care** | **Healthcare discipline(s)*** | **Pandemic phase** | **Hybrid implementation** | **In-person care interventions** | **Evaluation levels** |
| --- | --- | --- | --- | --- | --- | --- | --- | --- | --- |
| 1 | Abedin et al, 2025 | Germany | Observational | Secondary/Tertiary care | Gastrointestinal | Feb-Jun 2020 | Hybrid | Consultation | Reaction, Results |
| 2 | Angelovská et al, 2025 | Czech Republic | Mixed-methods | Primary care, Secondary/Tertiary care | General/Family medicine, Not specified | Feb-Jun 2020,Jul-Dec 2020,Jan-Jun 2021 | Only digital | N/A | Reaction |
| 3 | Albert et al, 2020 | Spain | Descriptive | Secondary/Tertiary care | Endocrinology | Feb-Jun 2020 | Only digital | N/A | Results |
| 4 | Altmann et al, 2022 | Austria | Experimental | Secondary/ Tertiary care | Neurology (medical) | Jul-Dec 2020,Jan-Jun 2021 | Only digital | N/A | Reaction |
| 5 | Ambrosini et al, 2020 | Italy | Descriptive | Secondary/ Tertiary care | Cancer (medical) | Feb-Jun 2020 | Hybrid | Consultation | Reaction |
| 6 | Amorim et al, 2021 | Portugal, Spain | Descriptive | Primary care, Secondary/ Tertiary care | General/Family medicine, Cardiology (medical) | Feb-Jun 2020,Jul-Dec 2020 | Only digital | N/A | No evaluation |
| 7 | Anderson et al, 2024 | United Kingdom | Qualitative study | Primary care | Nursing (primary care) | 2022 | Hybrid | Vital signs measurement, Laboratory tests, Physical examination | Reaction |
| 8 | Asomugha, 2025 | Sweden | Observational | Primary care | General/Family medicine | Feb-Jun 2020,Jul-Dec 2020,Jan-Jun 2021,Jul-Dec 2021,2022,2023-2024 | Only digital | N/A | No evaluation |
| 9 | Azzena et al, 2021 | Italy | Descriptive | Secondary/ Tertiary care | Plastic surgery/ burn unit | Feb-Jun 2020 | Only digital | N/A | No evaluation |
| 10 | Bailey and Nightingale, 2020 | United Kingdom | Descriptive | Secondary/ Tertiary care | Midwifery | Feb-Jun 2020,Jul-Dec 2020 | Hybrid | Imaging | No evaluation |
| 11 | Banks et al, 2021 | Ireland | Mixed-methods | Secondary/ Tertiary care | Neurology (medical) | Feb-Jun 2020 | Only digital | N/A | Reaction |
| 12 | Barsom et al, 2021 | The Netherlands | Mixed-methods | Secondary/ Tertiary care | General surgery, Gynaecology/Obstetrics (incl. reproductive care), Anaesthesiology | Feb-Jun 2020 | Hybrid | Not specified | Reaction |
| 13 | Barth et al, 2021 | Switzerland | Mixed-methods | Secondary/ Tertiary care | Cancer (medical), Gastrointestinal, Gynaecology/Obstetrics (incl. reproductive care) | Feb-Jun 2020 | Only digital | N/A | Reaction |
| 14 | Baumann et al, 2021 | France | Observational | Primary care | Midwifery | Feb-Jun 2020 | Not specified | N/A | No evaluation |
| 15 | Bauwens et al, 2023 | France | Observational | Secondary/ Tertiary care | Orthopedics and Trauma | Feb-Jun 2020 | Only digital | N/A | Results |
| 16 | Beauquis et al, 2021 | Belgium | Observational | Secondary/Tertiary care | Dentistry/ oral surgery | Feb-Jun 2020 | Hybrid | Minor procedures/ interventions, Consultation | Results |
| 17 | Békási et al, 2022 | Hungary | Observational | Community care | Internal medicine, General/Family medicine | Jan-Jun 2021,Jul-Dec 2021 | Only digital | N/A | Reaction |
| 18 | Benaque et al, 2020 | Spain | Descriptive | Secondary/ Tertiary care | Neurology (medical) | Feb-Jun 2020 | Only digital | N/A | Reaction |
| 19 | Berg and Wretborn, 2024 | Sweden | Observational | Primary care | Not specified | Feb-Jun 2020,Jul-Dec 2020,Jan-Jun 2021,Jul-Dec 2021,2022 | Only digital | N/A | Results |
| 20 | Berthelot et al, 2021 | France | Descriptive | Secondary/ Tertiary care | Cardiology (medical) | Feb-Jun 2020 | Only digital | N/A | No evaluation |
| 21 | Blackhall et al, 2020 | United Kingdom | Descriptive | Secondary/ Tertiary care | Maxillofacial surgery | Feb-Jun 2020 | Hybrid | Consultation, Minor procedures/ interventions, Surgical intervention | No evaluation |
| 22 | Bonalumi et al, 2020 | Italy | Descriptive | Secondary/ Tertiary care | Cardio/ thoracic (surgical),Vascular (surgical) | Feb-Jun 2020 | Hybrid | Surgical intervention | No evaluation |
| 23 | Bos et al, 2021 | The Netherlands | Descriptive | Secondary/ Tertiary care | Rheumatology | Feb-Jun 2020 | Hybrid | Minor procedures/ interventions, Therapy administration | Reaction |
| 24 | Bourdon et al, 2020 | France | Observational | Secondary/ Tertiary care | Ophthalmology | Feb-Jun 2020 | Hybrid | Consultation, Physical examination | Results |
| 25 | Boydell et al, 2021 | United Kingdom | Qualitative study | Secondary/ Tertiary care | Gynaecology/Obstetrics (incl. reproductive care) | Feb-Jun 2020,Jul-Dec 2020 | Only digital | N/A | Reaction |
| 26 | Brandes et al, 2020 | Italy | Descriptive | Secondary/ Tertiary care | Cancer (medical) | Feb-Jun 2020 | Hybrid | Consultation, Laboratory tests | No evaluation |
| 27 | Brunasso and Massone, 2020 | Italy | Descriptive | Secondary/ Tertiary care | Dermatology | Feb-Jun 2020 | Hybrid | Consultation | No evaluation |
| 28 | Burr et al, 2021 | United Kingdom | Qualitative study | Secondary/ Tertiary care | Endocrinology | Not specified | Only digital | N/A | No evaluation |
| 29 | Butler et al, 2020 | United Kingdom | Descriptive | Secondary/ Tertiary care | Cancer (surgical) | Feb-Jun 2020 | Hybrid | Surgical intervention, Consultation, Laboratory tests | No evaluation |
| 30 | Byrne and Watkinson, 2021 | United Kingdom | Descriptive | Secondary/ Tertiary care | Dentistry/ oral surgery | Feb-Jun 2020 | Only digital | N/A | Reaction |
| 31 | Capozzo et al, 2020 | Italy | Descriptive | Secondary/ Tertiary care | Neurology (medical) | Feb-Jun 2020 | Only digital | N/A | Reaction |
| 32 | Caravatta et al, 2020 | Italy | Descriptive | Secondary/ Tertiary care | Cancer (medical) | Feb-Jun 2020 | Hybrid | Consultation, Therapy administration | No evaluation |
| 33 | Carter et al, 2020 | United Kingdom | Observational | Secondary/ Tertiary care | Dentistry/ oral surgery | Feb-Jun 2020 | Hybrid | Consultation, Minor procedures/interventions | No evaluation |
| 34 | Casella et al, 2020 | Italy | Descriptive | Secondary/ Tertiary care | Cancer (surgical) | Feb-Jun 2020 | Hybrid | Consultation, Surgical intervention | No evaluation |
| 35 | Ceccato et al, 2021 | Italy | Observational | Secondary/ Tertiary care | Endocrinology | Feb-Jun 2020 | Hybrid | Minor procedures/interventions, Consultation, Imaging | No evaluation |
| 36 | Cervantes-Torres and Romero-Blanco, 2023 | Spain | Observational | Primary care | General/Family medicine | Feb-Jun 2020,Jul-Dec 2020,Jan-Jun 2021 | Only digital | N/A | Behavior, Results |
| 37 | Chana et al, 2022 | United Kingdom | Observational | Secondary/ Tertiary care | Vascular (surgical) | Feb-Jun 2020 | Hybrid | Consultation, Imaging, Minor procedures/interventions, Surgical intervention | No evaluation |
| 38 | Chappell et al, 2023 | United Kingdom | Observational | Primary care | General/Family medicine | Feb-Jun 2020,Jul-Dec 2020,Jan-Jun 2021,Jul-Dec 2021,2022 | Hybrid | Consultation | No evaluation |
| 39 | Chesser et al, 2021 | Multiple european (>5) | Descriptive | Secondary/ Tertiary care | Orthopedics & Trauma | Feb-Jun 2020 | Only digital | N/A | No evaluation |
| 40 | Chu et al, 2022 | Italy | Observational | Secondary/ Tertiary care | Cancer (surgical) | Feb-Jun 2020 | Only digital | N/A | No evaluation |
| 41 | Chukwusa et al, 2024 | United Kingdom | Observational | Primary care | General/Family medicine | Feb-Jun 2020,Jul-Dec 2020 | Only digital | N/A | No evaluation |
| 42 | Cohidon et al, 2022 | Switzerland | Mixed-methods | Primary care | General/Family medicine | Feb-Jun 2020,Jul-Dec 2020 | Only digital | N/A | No evaluation |
| 43 | Collins et al, 2021 | Ireland | Descriptive | Secondary/ Tertiary care | Urology | Feb-Jun 2020 | Hybrid | Consultation, Surgical intervention | Results |
| 44 | Corea et al, 2021 | Italy | Descriptive | Secondary/ Tertiary care | Neurology (medical) | Feb-Jun 2020 | Only digital | N/A | No evaluation |
| 45 | Crowley and Delargy, 2020 | Ireland | Descriptive | Community care, Primary care | General/Family medicine, Drug use/ Addiction services | Not specified | Hybrid | Laboratory tests, Therapy administration | No evaluation |
| 46 | Crusz et al, 2021 | United Kingdom | Descriptive | Secondary/ Tertiary care | Cancer (medical) | Feb-Jun 2020 | Hybrid | Consultation | No evaluation |
| 47 | Cuevas Fernández et al, 2024 | Spain | Observational | Primary care | General/Family medicine | Feb-Jun 2020,Jul-Dec 2020,Jan-Jun 2021,Jul-Dec 2021 | Hybrid | N/A | Results |
| 48 | Dambha-Miller et al, 2022 | United Kingdom | Observational | Primary care | General/Family medicine | Feb-Jun 2020,Jul-Dec 2020 | Only digital | N/A | No evaluation |
| 49 | De Marchi et al, 2021 | Italy | Observational | Secondary/ Tertiary care | Neurology (medical) | Feb-Jun 2020 | Hybrid | Consultation, in-patient interventions | Reaction |
| 50 | Del Hoyo et al, 2021 | Spain | Descriptive | Secondary/ Tertiary care | Gastrointestinal | Feb-Jun 2020 | Hybrid | Consultation | No evaluation |
| 51 | Deml et al, 2022 | Switzerland | Mixed-methods | Primary care | General/Family medicine | Feb-Jun 2020,Jul-Dec 2020,Jan-Jun 2021 | Not specified | Consultation | Reaction |
| 52 | Denti et al, 2024 | Italy | Observational | Secondary/Tertiary care | Cancer (medical),Cancer (surgical) | Jan-Jun 2021,Jul-Dec 2021,2022,2023-2024 | Hybrid | Physical examination (other),Minor procedures/interventions | Reaction, Results |
| 53 | Doica et al, 2021 | Romania | Experimental | Secondary/ Tertiary care, Primary care | Infectious diseases | Feb-Jun 2020 | Only digital | N/A | Reaction |
| 54 | Dunkerley et al, 2020 | United Kingdom | Descriptive | Secondary/ Tertiary care | Orthopedics & Trauma | Feb-Jun 2020 | Hybrid | Physical examination, Imaging, Minor procedures/interventions, Surgical intervention | Reaction |
| 55 | Dzakula et al, 2022 | Romania, Bulgaria, Croatia | Descriptive | Not specified | Not specified | Feb-Jun 2020,Jul-Dec 2020 | Not specified | Consultation | No evaluation |
| 56 | Eger et al, 2022 | Multiple european (>5) | Descriptive | Secondary/ Tertiary care | Respiratory | Jul-Dec 2020,Jan-Jun 2021 | Hybrid | Consultation | Reaction |
| 57 | Eide et al, 2023 | Norway | Observational | Primary care | General/Family medicine | Feb-Jun 2020,Jul-Dec 2020,Jan-Jun 2021,Jul-Dec 2021,2022 | Hybrid | Not specified | No evaluation |
| 58 | El Moazen et al, 2021 | Austria | Experimental | Secondary/ Tertiary care | Endocrinology | Feb-Jun 2020,Jul-Dec 2020 | Hybrid | Consultation | Results |
| 59 | Faria et al, 2020 | United Kingdom | Descriptive | Secondary/ Tertiary care | Orthopedics & Trauma | Feb-Jun 2020 | Hybrid | Surgical intervention, Physical examination, Minor procedures/interventions | No evaluation |
| 60 | Fieux et al, 2020 | France | Observational | Secondary/ Tertiary care | ENT / ORL (surgical) | Feb-Jun 2020 | Hybrid | Physical examination, Imaging, Consultation, Functional tests | Reaction |
| 61 | Florea et al, 2021 | Romania | Observational | Primary care | General/Family medicine | Feb-Jun 2020,Jul-Dec 2020 | Only digital | N/A | Reaction |
| 62 | Fonseca et al, 2024 | United Kingdom | Observational | Primary care | General/Family medicine | Feb-Jun 2020,Jul-Dec 2020,Jan-Jun 2021 | Only digital | N/A | No evaluation |
| 63 | Forrester et al, 2022 | Portugal | Mixed-methods | Primary care, Secondary/ Tertiary care | Cancer (medical) | Jul-Dec 2020,Jan-Jun 2021 | Only digital | N/A | No evaluation |
| 64 | Fuentes et al, 2020 | Spain | Descriptive | Secondary/ Tertiary care | Neurology (medical) | Feb-Jun 2020 | Only digital | N/A | No evaluation |
| 65 | Gabriel et al, 2021 | France | Descriptive | Secondary/ Tertiary care | Orthopedics & Trauma | Feb-Jun 2020 | Hybrid | Consultation | No evaluation |
| 66 | Garrido-Cumbrera et al, 2022 | Multiple european (>5) | Observational | Secondary/ Tertiary care, Primary care | Rheumatology | Feb-Jun 2020 | Not specified | Consultation | No evaluation |
| 67 | Gebbia et al, 2020 | Italy | Observational | Secondary/ Tertiary care | Cancer (medical) | Feb-Jun 2020 | Only digital | N/A | No evaluation |
| 68 | Geerdink et al, 2021 | The Netherlands | Experimental | Secondary/ Tertiary care | Orthopedics & Trauma | Feb-Jun 2020 | Hybrid | Consultation | Behavior, Results |
| 69 | Gesuete et al, 2023 | Italy | Observational | Secondary/ Tertiary care | Plastic surgery/ burn unit | Feb-Jun 2020,Jan-Jun 2021,Jul-Dec 2021 | Only digital | N/A | Reaction |
| 70 | Gilbert et al , 2020 | United Kingdom | Descriptive | Secondary/ Tertiary care | Orthopedics & Trauma | Feb-Jun 2020 | Hybrid | Consultation | Reaction |
| 71 | Giudice et al, 2020 | Italy | Descriptive | Secondary/ Tertiary care, Primary care | Dentistry/ oral surgery | Feb-Jun 2020 | Hybrid | Surgical intervention, Consultation | Behavior |
| 72 | Gleeson et al, 2022 | Ireland | Mixed-methods | Primary care | General/Family medicine, Pharmacy | Jan-Jun 2021,Jul-Dec 2021 | Only digital | N/A | Reaction |
| 73 | Gomes-de Almeida et al, 2021 | Portugal | Observational | Primary care | General/Family medicine | Feb-Jun 2020 | Only digital | N/A | Reaction |
| 74 | Gonzalez et al, 2020 | United Kingdom | Descriptive | Secondary/ Tertiary care | Gastrointestinal | Feb-Jun 2020 | Only digital | N/A | No evaluation |
| 75 | Grobe-Einsler et al, 2021 | Germany | Observational | Secondary/ Tertiary care | Neurology (medical) | Not specified | Only digital | N/A | Results |
| 76 | Hardman et al, 2021 | United Kingdom | Observational | Secondary/Tertiary care | Cancer (surgical) | Feb-Jun 2020,Jul-Dec 2020 | Hybrid | Consultation | Results |
| 77 | Hartl et al, 2021 | Austria | Observational | Secondary/ Tertiary care | Gastrointestinal | Feb-Jun 2020 | Hybrid | Consultation | Reaction |
| 78 | Indini et al, 2020 | Italy | Observational | Secondary/ Tertiary care | Cancer (medical) | Feb-Jun 2020 | Not specified | N/A | No evaluation |
| 79 | Jacome et al, 2022 | Portugal, Spain | Observational | Secondary/ Tertiary care | Immunology, Respiratory | Feb-Jun 2020 | Hybrid | Consultation | No evaluation |
| 80 | Johansson et al, 2025 | Finland | Observational | Primary care | General/Family medicine | Feb-Jun 2020,Jul-Dec 2020,Jan-Jun 2021,Jul-Dec 2021,2022 | Only digital | N/A | No evaluation |
| 81 | Johnsen et al, 2021 | Norway | Observational | Primary care | General/Family medicine | Feb-Jun 2020 | Hybrid | Consultation | Reaction |
| 82 | Joy et al, 2020 | United Kingdom | Observational | Primary care | General/Family medicine | Feb-Jun 2020 | Hybrid | Consultation | No evaluation |
| 83 | Kaddour et al, 2022 | United Kingdom | Descriptive | Secondary/Tertiary care | Cancer (surgical) | Feb-Jun 2020,Jul-Dec 2020 | Only digital | N/A | No evaluation |
| 84 | Kenis et al, 2022 | Belgium | Qualitative study | Secondary/ Tertiary care | Cancer (medical) | Feb-Jun 2020,Jul-Dec 2020 | Hybrid | Laboratory tests, Consultation | No evaluation |
| 85 | Kilduff et al, 2020 | United Kingdom | Observational | Secondary/ Tertiary care | Ophthalmology | Feb-Jun 2020 | Hybrid | Physical examination, Consultation | No evaluation |
| 86 | King et al, 2024 | United Kingdom | Qualitative study | Primary care | General/Family medicine | 2022,Feb-Jun 2020 | Hybrid | Consultation, Physical examination, Therapy administration | No evaluation |
| 87 | Kirk et al, 2024 | Denmark | Observational | Primary care | General/Family medicine | Feb-Jun 2020,Jul-Dec 2020,Jan-Jun 2021,Jul-Dec 2021 | Only digital | N/A | No evaluation |
| 88 | Klain et al, 2021 | Italy | Observational | Secondary/ Tertiary care | Cancer (surgical) | Feb-Jun 2020 | Hybrid | Imaging, Consultation | Reaction |
| 89 | Kollmann et al, 2024 | The Netherlands | Qualitative study | Primary care | General/Family medicine | Feb-Jun 2020,Jul-Dec 2020 | Only digital | N/A | Reaction |
| 90 | Krawczyk et al, 2024 | Poland | Observational | Secondary/Tertiary care | Multiple (>5) | Feb-Jun 2020,Jul-Dec 2020,Jan-Jun 2021,Jul-Dec 2021,2022 | Only digital | N/A | No evaluation |
| 91 | Lapão et al, 2021 | Portugal | Descriptive | Primary care | General/Family medicine | Feb-Jun 2020,Jul-Dec 2020 | Only digital | N/A | Reaction |
| 92 | Legrottaglie et al, 2021 | Italy | Observational | Secondary/ Tertiary care | Ophthalmology | Feb-Jun 2020 | Hybrid | Physical examination, Minor procedures/interventions | No evaluation |
| 93 | Li et al, 2021 | United Kingdom | Observational | Secondary/Tertiary care | Ophthalmology | Feb-Jun 2020 | Hybrid | Consultation | Reaction, Results |
| 94 | Loezar-Hernández et al, 2023 | Spain | Qualitative study | Primary care | Midwifery | Jul-Dec 2021,2022 | Only digital | N/A | No evaluation |
| 95 | Luciani et al, 2020 | Italy | Descriptive | Secondary/ Tertiary care | Urology | Feb-Jun 2020 | Hybrid | Consultation | No evaluation |
| 96 | Luengo-Alonso et al, 2020 | Spain | Observational | Secondary/ Tertiary care | Orthopedics & Trauma | Feb-Jun 2020 | Hybrid | Consultation, Surgical intervention | Reaction |
| 97 | Mackintosh et al, 2023 | United Kingdom | Observational | Secondary/ Tertiary care | Renal (medical) | Feb-Jun 2020,Jul-Dec 2020,Jan-Jun 2021,Jul-Dec 2021 | Hybrid | Surgical intervention, Consultation | No evaluation |
| 98 | Manzi et al, 2022 | Italy | Descriptive | Secondary/ Tertiary care | Cardiology (medical) | Feb-Jun 2020 | Hybrid | Consultation, In-patient interventions | No evaluation |
| 99 | Mathai et al, 2020 | United Kingdom | Descriptive | Secondary/ Tertiary care | Orthopedics & Trauma | Feb-Jun 2020 | Hybrid | Surgical intervention, Consultation, Vital signs measurement, In-patient interventions, Imaging | No evaluation |
| 100 | Mazzone et al, 2020 | Italy | Descriptive | Secondary/ Tertiary care | Cardiology (medical) | Feb-Jun 2020 | Hybrid | Minor procedures/interventions, In-patient interventions, Therapy administration, Imaging | No evaluation |
| 101 | McKenna et al, 2020 | Ireland | Observational | Secondary/ Tertiary care | Neurology (medical) | Feb-Jun 2020 | Only digital | N/A | Reaction |
| 102 | Miceli et al, 2022 | Italy | Descriptive | Secondary/ Tertiary care | Cancer (medical) | Not specified | Hybrid | Consultation | No evaluation |
| 103 | Miguela Alvarez et al, 2021 | Spain | Descriptive | Secondary/ Tertiary care | Orthopedics & Trauma | Feb-Jun 2020 | Hybrid | Consultation | No evaluation |
| 104 | Minghelli et al, 2020 | Portugal | Descriptive | Primary care | Physiotherapy | Feb-Jun 2020 | Hybrid | Consultation | No evaluation |
| 105 | Minniti et al, 2020 | Italy | Observational | Secondary/ Tertiary care | Rheumatology | Feb-Jun 2020 | Hybrid | Therapy administration | No evaluation |
| 106 | Montesi et al, 2020 | Italy | Descriptive | Secondary/ Tertiary care | Cancer (medical) | Feb-Jun 2020 | Hybrid | Therapy administration | No evaluation |
| 107 | Morgan et al, 2020 | United Kingdom | Descriptive | Secondary/ Tertiary care | Orthopedics & Trauma | Feb-Jun 2020 | Hybrid | Surgical intervention, Consultation, Imaging | No evaluation |
| 108 | Morreel et al, 2020 | Belgium | Observational | Primary care | General/Family medicine | Feb-Jun 2020 | Hybrid | Consultation | No evaluation |
| 109 | Motolese et al, 2020 | Italy | Observational | Secondary/ Tertiary care | Neurology (medical) | Feb-Jun 2020 | Only digital | N/A | Reaction, Behavior |
| 110 | Mugnai et al, 2021 | Italy | Observational | Secondary/ Tertiary care | Cardiology (medical) | Feb-Jun 2020 | Hybrid | Consultation | Results |
| 111 | Munda, 2023 | Slovenia | Observational | Secondary/Tertiary care | Endocrinology | Feb-Jun 2020,Jul-Dec 2020 | Hybrid | Consultation | Results |
| 112 | Murphy et al, 2021 | United Kingdom | Mixed-methods | Primary care | General/Family medicine | Feb-Jun 2020,Jul-Dec 2020 | Hybrid | Consultation | Reaction |
| 113 | Murris et al, 2021 | France | Descriptive | Secondary/ Tertiary care | Cancer (surgical) | Feb-Jun 2020 | Hybrid | Consultation, Surgical intervention, Therapy administration | No evaluation |
| 114 | Muschol et al, 2023 | Germany | Experimental | Secondary/ Tertiary care | Orthopedics & Trauma | Jul-Dec 2020,Jan-Jun 2021 | Only digital | Consultation | Reaction, Results |
| 115 | Muthiah et al, 2023 | United Kingdom | Observational | Secondary/Tertiary care | Dermatology | Feb-Jun 2020 | Hybrid | Consultation | Reaction, Results |
| 116 | Nebsbjerg et al, 2024 | Denmark | Observational | Primary care | General/Family medicine | Feb-Jun 2020,Jul-Dec 2020,Jan-Jun 2021,Jul-Dec 2021 | Only digital | N/A | No evaluation |
| 117 | Newby et al, 2024 | United Kingdom | Observational | Community care | Exercise referral program | Feb-Jun 2020,Jul-Dec 2020,Jan-Jun 2021,Jul-Dec 2021 | Hybrid | Therapy administration | Reaction, Results |
| 118 | Nielsen and Andersen, 2025 | Denmark | Observational | Primary care | General/Family medicine | Feb-Jun 2020,Jul-Dec 2020,Jan-Jun 2021 | Hybrid | Consultation | No evaluation |
| 119 | Noé et al, 2021 | Spain | Observational | Secondary/ Tertiary care | Neurology (medical) | Feb-Jun 2020 | Only digital | N/A | Reaction, Results |
| 120 | Nune et al, 2020 | United Kingdom | Observational | Secondary/ Tertiary care | Rheumatology | Feb-Jun 2020 | Hybrid | Consultation, Laboratory tests, Therapy administration | No evaluation |
| 121 | Nuñez et al, 2020 | Spain | Observational | Secondary/ Tertiary care | Orthopedics & Trauma | Feb-Jun 2020 | Hybrid | Consultation, Surgical intervention, Imaging | No evaluation |
| 122 | O'Donovan et al, 2020 | Ireland | Descriptive | Secondary/ Tertiary care | Haematology, Dentistry/ oral surgery, Physiotherapy | Feb-Jun 2020 | Hybrid | Consultation | Reaction, Results |
| 123 | Ochieng et al, 2022 | United Kingdom | Qualitative study | Home/ Residential care, Primary care | General/Family medicine | Feb-Jun 2020 | Only digital | N/A | Reaction |
| 124 | Ohnleiter et al, 2020 | France | Descriptive | Secondary/ Tertiary care | Cancer (medical) | Feb-Jun 2020 | Hybrid | Therapy administration, Consultation, Imaging | No evaluation |
| 125 | Omboni, et al, 2021 | Italy | Observational | Primary care | General/Family medicine | Feb-Jun 2020,Jul-Dec 2020 | Hybrid | Functional tests, Vital signs measurement | Reaction |
| 126 | Paleri et al, 2020 | United Kingdom | Descriptive | Secondary/ Tertiary care | Cancer (surgical) | Feb-Jun 2020 | Hybrid | Consultation | Reaction |
| 127 | Papoutsi et al, 2022 | United Kingdom | Qualitative study | Primary care | General/Family medicine | Feb-Jun 2020,Jul-Dec 2020,Jan-Jun 2021 | Only digital | N/A | Reaction |
| 128 | Pareyson et al , 2021 | Italy | Descriptive | Secondary/ Tertiary care | Neurology (medical) | Feb-Jun 2020,Jul-Dec 2020 | Only digital | N/A | Reaction |
| 129 | Pascual et al, 2023 | Spain | Descriptive | Secondary/ Tertiary care | Multiple surgical (>5) | Not specified | Hybrid | Consultation, Laboratory tests, Minor procedures/interventions, Surgical intervention | No evaluation |
| 130 | Paudyal et al, 2021 | Multiple european (>5) | Qualitative study | Primary care, Secondary/ Tertiary care | Pharmacy | Feb-Jun 2020,Jul-Dec 2020 | Only digital | N/A | No evaluation |
| 131 | Peretto et al, 2020 | Italy | Descriptive | Secondary/ Tertiary care | Cardiology (medical) | Feb-Jun 2020,Jul-Dec 2020 | Hybrid | In-patient interventions, Minor procedures/interventions, Functional tests, Laboratory tests | Reaction |
| 132 | Perrin et al, 2023 | France | Observational | Secondary/ Tertiary care | Orthopedics & Trauma | Feb-Jun 2020 | Hybrid | Consultation | Reaction |
| 133 | Perrone et al, 2021 | Italy | Observational | Secondary/ Tertiary care | Cancer (surgical) | Feb-Jun 2020 | Hybrid | Imaging, Therapy administration, Surgical intervention | No evaluation |
| 134 | Phillips et al, 2022 | United Kingdom | Descriptive | Secondary/ Tertiary care | Plastic surgery/ burn unit | Feb-Jun 2020 | Hybrid | Consultation | No evaluation |
| 135 | Piane et al, 2022 | Italy | Observational | Secondary/ Tertiary care, Primary care | Cancer (medical),Cardiology (medical),Endocrinology, Cancer (surgical),General/Family medicine | Feb-Jun 2020,Jul-Dec 2020,Jan-Jun 2021 | Not specified | Not specified | No evaluation |
| 136 | Pignatti et al, 2020 | Italy | Descriptive | Secondary/ Tertiary care | Plastic surgery/ burn unit | Feb-Jun 2020 | Hybrid | Surgical intervention, Consultation | No evaluation |
| 137 | Pinar et al, 2020 | France | Observational | Secondary/ Tertiary care | Urology | Feb-Jun 2020 | Only digital | N/A | Reaction |
| 138 | Piro et al, 2020 | Italy | Observational | Secondary/ Tertiary care | Cardiology (medical) | Feb-Jun 2020 | Hybrid | Consultation, In-patient interventions | Reaction, Results |
| 139 | Pulvirenti et al, 2020 | Italy | Observational | Secondary/ Tertiary care | Immunology | Feb-Jun 2020 | Only digital | N/A | Results |
| 140 | Quinn et al, 2021 | United Kingdom | Observational | Secondary/ Tertiary care | Gynaecology/Obstetrics (incl. reproductive care) | Feb-Jun 2020 | Only digital | N/A | Reaction |
| 141 | Rajasekaran et al, 2020 | United Kingdom | Descriptive | Secondary/ Tertiary care | Cancer (surgical) | Feb-Jun 2020 | Hybrid | Consultation, Minor procedures/interventions, Surgical intervention, Therapy administration | No evaluation |
| 142 | Ramaswami et al, 2021 | United Kingdom | Descriptive | Secondary/ Tertiary care | Rare/Genetic diseases (Other) | Feb-Jun 2020,Jul-Dec 2020,Jan-Jun 2021 | Hybrid | Consultation, Laboratory tests, Imaging, Functional tests, Therapy administration | No evaluation |
| 143 | Rayo et al, 2022 | Spain | Descriptive | Secondary/ Tertiary care | Gynaecology/Obstetrics (incl. reproductive care) | Feb-Jun 2020 | Hybrid | Consultation, Imaging, Laboratory tests | No evaluation |
| 144 | Riboli-Sasco et al, 2024 | United Kingdom | Qualitative study | Primary care | General/Family medicine | Feb-Jun 2020,Jul-Dec 2020, Jul-Dec 2021,Jan-Jun 2021 | Only digital | N/A | Reaction |
| 145 | Rimmer and Al Wattar, 2020 | United Kingdom | Observational | Secondary/ Tertiary care | Gynaecology/Obstetrics (incl. reproductive care) | Feb-Jun 2020 | Hybrid | Consultation | No evaluation |
| 146 | Rodler et al, 2020 | Germany | Observational | Secondary/ Tertiary care | Cancer (surgical) | Feb-Jun 2020 | Hybrid | Therapy administration | Reaction |
| 147 | Romano et al, 2021 | Italy | Descriptive | Community care | Physiotherapy | Feb-Jun 2020 | Only digital | N/A | No evaluation |
| 148 | Rossi et al, 2020 | Italy | Descriptive | Secondary/ Tertiary care | Cancer (medical) | Feb-Jun 2020 | Hybrid | Consultation, Surgical intervention | No evaluation |
| 149 | Runfola et al, 2020 | Italy | Observational | Secondary/ Tertiary care | General surgery | Feb-Jun 2020 | Only digital | N/A | No evaluation |
| 150 | Russo et al, 2021 | Italy | Observational | Secondary/ Tertiary care | Cardiology (medical) | Feb-Jun 2020 | Only digital | N/A | Reaction |
| 151 | Rzewuska et al, 2024 | United Kingdom | Mixed-methods | Secondary/Tertiary care | Palliative care/end-of-life, Gastrointestinal, Dermatology | Feb-Jun 2020,Jul-Dec 2020,Jan-Jun 2021,Jul-Dec 2021,2022 | Hybrid | Consultation | Reaction |
| 152 | Sacchi et al, 2023 | Sweden | Mixed-methods | Secondary/ Tertiary care | Cardiology (medical) | Jul-Dec 2020,Jan-Jun 2021 | Hybrid | Consultation | Behavior |
| 153 | Saibeni et al, 2020 | Italy | Descriptive | Secondary/ Tertiary care | Gastrointestinal | Feb-Jun 2020 | Hybrid | Therapy administration, E ndoscopic procedures, Imaging | No evaluation |
| 154 | Saint-Lary et al, 2020 | France | Observational | Primary care | General/Family medicine | Feb-Jun 2020 | Not specified | N/A | No evaluation |
| 155 | Salzano et al, 2020 | Italy | Observational | Secondary/ Tertiary care | Cardiology (medical) | Feb-Jun 2020 | Only digital | N/A | Results |
| 156 | Sanders et al, 2025 | United Kingdom | Observational | Alternative medicine | Chiropractic | Feb-Jun 2020 | Only digital | N/A | Reaction |
| 157 | Scaldaferri et al, 2020 | Italy | Descriptive | Secondary/ Tertiary care | Gastrointestinal | Feb-Jun 2020 | Hybrid | Consultation, Therapy administration, Endoscopic procedures | No evaluation |
| 158 | Schnoor et al, 2023 | The Netherlands | Observational | Primary care | General/Family medicine | Jan-Jun 2021,Jul-Dec 2021,2022 | Hybrid | Laboratory tests | Reaction |
| 159 | Severino et al, 2022 | Italy | Observational | Secondary/ Tertiary care | Cardiology (medical) | Feb-Jun 2020,Jul-Dec 2020,Jan-Jun 2021 | Only digital | N/A | Results |
| 160 | Shah et al, 2020 | United Kingdom | Descriptive | Secondary/ Tertiary care | Dentistry/ oral surgery | Feb-Jun 2020 | Hybrid | Consultation, Surgical intervention, In-patient interventions, Physical examination | No evaluation |
| 161 | Shaw et al, 2021 | United Kingdom | Mixed-methods | Primary care, Secondary/ Tertiary care | Not specified | Feb-Jun 2020,Jul-Dec 2020,Jan-Jun 2021,Jul-Dec 2021 | Not specified | N/A | Reaction, Behavior |
| 162 | Sheil and McAuliffe, 2021 | Ireland | Descriptive | Secondary/ Tertiary care | Gynaecology/Obstetrics (incl. reproductive care),Midwifery | Feb-Jun 2020 | Hybrid | Consultation, Imaging | No evaluation |
| 163 | Silsand et al, 2021 | Norway | Qualitative study | Secondary/ Tertiary care, Primary care | Geriatry/ Elderly care,General/Family medicine | Feb-Jun 2020 | Only digital | N/A | Reaction |
| 164 | Smrke et al, 2020 | United Kingdom | Descriptive | Secondary/ Tertiary care | Cancer (medical) | Feb-Jun 2020 | Hybrid | Therapy administration, Consultation | Reaction |
| 165 | Solans et al, 2021 | Spain | Observational | Primary care | General/Family medicine | Feb-Jun 2020 | Hybrid | Not specified | No evaluation |
| 166 | Solberg Carlsson et al, 2023 | Sweden | Qualitative study | Primary care | General/Family medicine | Feb-Jun 2020,Jul-Dec 2020,Jan-Jun 2021,Jul-Dec 2021 | Only digital | N/A | Reaction |
| 167 | Soler et al, 2020 | Spain | Observational | Secondary/ Tertiary care | Renal (medical) | Feb-Jun 2020 | Hybrid | Minor procedures/interventions, Laboratory tests, Surgical intervention | No evaluation |
| 168 | Somani et al, 2020 | United Kingdom | Descriptive | Secondary/ Tertiary care | Urology | Feb-Jun 2020 | Hybrid | Minor procedures/interventions, Endoscopic procedures, Surgical intervention | No evaluation |
| 169 | Stansfield et al, 2021 | United Kingdom | Observational | Secondary/ Tertiary care | ENT / ORL (surgical) | Feb-Jun 2020 | Hybrid | Minor procedures/interventions | No evaluation |
| 170 | Stepaniuk et al, 2022 | Poland | Observational | Secondary/ Tertiary care, Primary care | Dermatology, General/Family medicine,Internal medicine | Feb-Jun 2020,Jul-Dec 2020 | Not specified | N/A | Reaction |
| 171 | Stewart et al, 2022 | United Kingdom | Mixed-methods | Primary care | General/Family medicine | Not specified | Only digital | N/A | Reaction, Behavior |
| 172 | Swierad et al, 2020 | Poland | Observational | Secondary/ Tertiary care | Cardiology (medical),Cardio/ thoracic (surgical) | Feb-Jun 2020 | Hybrid | Consultation, Functional tests | No evaluation |
| 173 | Tensen et al, 2023 | The Netherlands | Descriptive | Primary care | General/Family medicine | Feb-Jun 2020,Jul-Dec 2020,Jan-Jun 2021,Jul-Dec 2021 | Hybrid | N/A | Reaction |
| 174 | Testa et al, 2022 | Italy | Descriptive | Secondary/ Tertiary care | Cardiology (medical),Endocrinology,Occupational medicine | Feb-Jun 2020,Jul-Dec 2020 | Hybrid | Consultation, Laboratory tests | Reaction, Behavior |
| 175 | Thomas et al, 2020 | United Kingdom | Descriptive | Secondary/ Tertiary care | Immunology | Feb-Jun 2020 | Hybrid | Consultation, Physical examination, Laboratory tests | Reaction |
| 176 | Tortajada-Goitia et al, 2020 | Spain | Descriptive | Secondary/ Tertiary care | Pharmacy | Feb-Jun 2020 | Not specified | N/A | No evaluation |
| 177 | Tyler et al, 2021 | United Kingdom | Observational | Secondary/ Tertiary care | Multiple (>5) | Feb-Jun 2020,Jul-Dec 2020 | Hybrid | Consultation | Reaction |
| 178 | van de Haar et al, 2020 | Multiple european (>5) | Descriptive | Secondary/ Tertiary care | Cancer (medical),Cancer (surgical) | Feb-Jun 2020 | Hybrid | Physical examination, Consultation | No evaluation |
| 179 | van de Vijver et al, 2022 | The Netherlands | Qualitative study | Primary care | General/Family medicine | Feb-Jun 2020,Jul-Dec 2020,Jan-Jun 2021 | Only digital | N/A | Reaction |
| 180 | Vassallo et al, 2024 | Malta | Descriptive | Primary care | General/Family medicine | Jul-Dec 2021 | Hybrid | Consultation | No evaluation |
| 181 | Vasta et al, 2021 | Italy | Descriptive | Secondary/ Tertiary care | Neurology (medical) | Feb-Jun 2020 | Only digital | N/A | Reaction |
| 182 | Viegas et al, 2023 | France | Observational | Primary care | General/Family medicine | Jan-Jun 2021,Jul-Dec 2021 | Hybrid | Consultation | No evaluation |
| 183 | Wanat et al, 2021 | Multiple european (>5) | Qualitative study | Primary care | General/Family medicine | Feb-Jun 2020,Jul-Dec 2020 | Hybrid | Consultation | No evaluation |
| 184 | Wherton et al, 2021 | United Kingdom | Mixed-methods | Primary care, Secondary/ Tertiary care, Community care | Multiple (>5) | Feb-Jun 2020,Jul-Dec 2020 | Hybrid | Physical examination, Laboratory tests | Reaction, Behavior |
| 185 | Wilk et al, 2021 | Poland | Observational | Secondary/Tertiary care | Endocrinology | Feb-Jun 2020 | Hybrid | Consultation | Reaction, Results |
| 186 | Willems et al, 2020 | Germany | Observational | Secondary/ Tertiary care | Neurology (medical) | Feb-Jun 2020 | Hybrid | Consultation | Reaction |
| 187 | Wu et al, 2021 | United Kingdom | Observational | Secondary/ Tertiary care | Cancer (medical),Cancer (surgical) | Feb-Jun 2020,Jul-Dec 2020 | Only digital | N/A | Reaction, Results |
| 188 | Zakrzewski et al, 2025 | Poland | Observational | Primary care | General/Family medicine | Feb-Jun 2020,Jul-Dec 2020,Jan-Jun 2021,Jul-Dec 2021,2022,2023-2024 | Only digital | N/A | No evaluation |
| 189 | Zondag et al, 2025 | The Netherlands | Observational | Secondary/Tertiary care | Multiple (>5) | Feb-Jun 2020,Jul-Dec 2020,Jan-Jun 2021,Jul-Dec 2021,2022 | Hybrid | Consultation | Results |

***Healthcare disciplines categorized under ‘other’ in main article text include: cardiothoracic surgery (n=2), ear, nose and throat (n=2), general surgery (n=2), internal medicine (n=2), nephrology (n=2), respiratory medicine (n=2), vascular surgery (n=2), addiction services (n=1), anesthesiology (n=1), chiropractic (n=1), exercise program (n=1), geriatry (n=1), hematology (n=1), infectious diseases (n=1), maxillofacial surgery (n=1), nursing (primary care) (n=1), occupational medicine (n=1), pain management (n=1), rare/ genetic diseases (n=1).

**References**

1. Abedin N, Kilbinger C, Queck A, Weiler N, Pathil A, Mihm U, et al. Telemedicine Hybrid Care Models in Gastroenterology Outpatient Care: Results from a German Tertiary Center. J Clin Med. 2025 Apr 4;14(7). PMID: 40217919. doi: 10.3390/jcm14072471.

2. Albert L, Capel I, Garcia-Saez G, Martin-Redondo P, Hernando ME, Rigla M. Managing gestational diabetes mellitus using a smartphone application with artificial intelligence (SineDie) during the COVID-19 pandemic: Much more than just telemedicine. Diabetes Res Clin Pract. 2020 Nov;169:108396. PMID: 32890548. doi: 10.1016/j.diabres.2020.108396.

3. Altmann P, Leutmezer F, Ponleitner M, Ivkic D, Krajnc N, Rommer PS, et al. Remote visits for people with multiple sclerosis during the COVID-19 pandemic in Austria: The TELE MS randomized controlled trial. Digit Health. 2022 Jan-Dec;8:20552076221112154. PMID: 35847524. doi: 10.1177/20552076221112154.

4. Ambrosini F, Di Stasio A, Mantica G, Cavallone B, Serao A. COVID-19 pandemic and uro-oncology follow-up: A "virtual" multidisciplinary team strategy and patients' satisfaction assessment. Arch Ital Urol Androl. 2020 Jun 23;92(2). PMID: 32597103. doi: 10.4081/aiua.2020.2.78.

5. Amorim P, Brito D, Castelo-Branco M, Fabrega C, Gomes da Costa F, Martins H, et al. Telehealth Opportunities in the COVID-19 Pandemic Early Days: What Happened, Did Not Happen, Should Have Happened, and Must Happen in the Near Future? Telemed J E Health. 2021 Oct;27(10):1194-9. PMID: 33264071. doi: 10.1089/tmj.2020.0386.

6. Anderson H, Scantlebury A, Galdas P, Adamson J. Remote and technology-mediated working during the COVID-19 pandemic: A qualitative exploration of the experiences of nurses working in general practice (the GenCo Study). J Adv Nurs. 2024 Apr;80(4):1592-606. PMID: 37909600. doi: 10.1111/jan.15921.

7. Angelovska O, Dobiasova K, Tesinova JK. Pandemic COVID-19 as a challenge for telemedicine in the Czech Republic. Int J Health Plann Manage. 2025 Jan;40(1):271-86. PMID: 39497023. doi: 10.1002/hpm.3863.

8. Asomugha AU, Pakai A. Trends and Shifts in Swedish Telemedicine Consultations During the Pre-COVID-19, COVID-19, and Post-COVID-19 Periods: Retrospective Observational Study. JMIR Form Res. 2025 May 16;9:e60294. PMID: 40378415. doi: 10.2196/60294.

9. Azzena B, Perozzo FAG, De Lazzari A, Valotto G, Pontini A. Burn Unit admission and management protocol during COVID-19 pandemic. Burns. 2021 Feb;47(1):52-7. PMID: 33148487. doi: 10.1016/j.burns.2020.09.004.

10. Bailey E, Nightingale S. Navigating maternity service redesign in a global pandemic: A report from the field. Midwifery. 2020 Oct;89:102780. PMID: 32570094. doi: 10.1016/j.midw.2020.102780.

11. Banks J, Corrigan D, Grogan R, El-Naggar H, White M, Doran E, et al. LoVE in a time of CoVID: Clinician and patient experience using telemedicine for chronic epilepsy management. Epilepsy Behav. 2021 Feb;115:107675. PMID: 33342712. doi: 10.1016/j.yebeh.2020.107675.

12. Barsom EZ, Meijer HAW, Blom J, Schuuring MJ, Bemelman WA, Schijven MP. Emergency upscaling of video consultation during the COVID-19 pandemic: Contrasting user experience with data insights from the electronic health record in a large academic hospital. Int J Med Inform. 2021 Jun;150:104463. PMID: 33872824. doi: 10.1016/j.ijmedinf.2021.104463.

13. Barth J, Canella C, Oehler M, Witt CM. Digital Consultations During COVID-19: A Multiperspective Mixed-Methods Study in an Integrative Medicine Setting in Switzerland. J Altern Complement Med. 2021 Jul;27(7):569-78. PMID: 33960805. doi: 10.1089/acm.2020.0539.

14. Baumann S, Gaucher L, Bourgueil Y, Saint-Lary O, Gautier S, Rousseau A. Adaptation of independent midwives to the COVID-19 pandemic: A national descriptive survey. Midwifery. 2021 Mar;94:102918. PMID: 33418511. doi: 10.1016/j.midw.2020.102918.

15. Bauwens PH, Fayard JM, Tatar M, Abid H, Freychet B, Sonnery-Cottet B, et al. Evaluation of a smartphone application for self-rehabilitation after anterior cruciate ligament reconstruction during a COVID-19 lockdown. Orthop Traumatol Surg Res. 2023 Feb;109(1):103342. PMID: 35660080. doi: 10.1016/j.otsr.2022.103342.

16. Beauquis J, Petit AE, Michaux V, Sague V, Henrard S, Leprince JG. Dental Emergencies Management in COVID-19 Pandemic Peak: A Cohort Study. J Dent Res. 2021 Apr;100(4):352-60. PMID: 33541180. doi: 10.1177/0022034521990314.

17. Bekasi S, Girasek E, Gyorffy Z. Telemedicine in community shelters: possibilities to improve chronic care among people experiencing homelessness in Hungary. Int J Equity Health. 2022 Dec 17;21(1):181. PMID: 36528777. doi: 10.1186/s12939-022-01803-4.

18. Benaque A, Gurruchaga MJ, Abdelnour C, Hernandez I, Canabate P, Alegret M, et al. Dementia Care in Times of COVID-19: Experience at Fundacio ACE in Barcelona, Spain. J Alzheimers Dis. 2020;76(1):33-40. PMID: 32538856. doi: 10.3233/JAD-200547.

19. Berg J, Wretborn J. Impact of the COVID-19 pandemic on the National Telehealth Service for triage and referral in Sweden: a national retrospective observational study. BMJ Open. 2024 Dec 4;14(12):e091627. PMID: 39632112. doi: 10.1136/bmjopen-2024-091627.

20. Berthelot E, Flécher E, Roubille F, Damy T, Lamblin N. Impact of the COVID-19 pandemic on the burden of chronic heart failure patients in France. Ann Cardiol Angeiol (Paris). 2021 Oct;70(4):191-5. PMID: 34517972. doi: 10.1016/j.ancard.2021.07.002.

21. Blackhall KK, Downie IP, Ramchandani P, Kusanale A, Walsh S, Srinivasan B, et al. Provision of Emergency Maxillofacial Service During the COVID-19 Pandemic: A Collaborative Five Centre UK Study. Br J Oral Maxillofac Surg. 2020 Jul;58(6):698-703. PMID: 32482348. doi: 10.1016/j.bjoms.2020.05.020.

22. Bonalumi G, Giambuzzi I, Barbone A, Ranieri C, Cavallotti L, Trabattoni P, et al. A call to action becomes practice: cardiac and vascular surgery during the COVID-19 pandemic based on the Lombardy emergency guidelines. Eur J Cardiothorac Surg. 2020 Aug 1;58(2):319-27. PMID: 32584978. doi: 10.1093/ejcts/ezaa204.

23. Bos WH, van Tubergen A, Vonkeman HE. Telemedicine for patients with rheumatic and musculoskeletal diseases during the COVID-19 pandemic; a positive experience in the Netherlands. Rheumatol Int. 2021 Mar;41(3):565-73. PMID: 33449162. doi: 10.1007/s00296-020-04771-6.

24. Bourdon H, Jaillant R, Ballino A, El Kaim P, Debillon L, Bodin S, et al. Teleconsultation in primary ophthalmic emergencies during the COVID-19 lockdown in Paris: Experience with 500 patients in March and April 2020. J Fr Ophtalmol. 2020 Sep;43(7):577-85. PMID: 32564983. doi: 10.1016/j.jfo.2020.05.005.

25. Boydell N, Reynolds-Wright JJ, Cameron ST, Harden J. Women's experiences of a telemedicine abortion service (up to 12 weeks) implemented during the coronavirus (COVID-19) pandemic: a qualitative evaluation. BJOG. 2021 Oct;128(11):1752-61. PMID: 34138505. doi: 10.1111/1471-0528.16813.

26. Brandes AA AA, Artioli F, Cappuzzo F, Cavanna L, Frassineti GL, Frassoldati A, Leonardi F, Longo G, Maestri A, Tassinari D, Franceschi E, Di Nunno V, Pinto C. Fighting cancer in coronavirus disease era: organization of work in medical oncology departments in Emilia Romagna region of Italy. Future Oncol. 2020;16(20).

27. Brunasso AMG, Massone C. Teledermatologic monitoring for chronic cutaneous autoimmune diseases with smartworking during COVID-19 emergency in a tertiary center in Italy. Dermatol Ther. 2020 Jul;33(4):e13495. PMID: 32458588. doi: 10.1111/dth.13695.

28. Burr O, Berry A, Joule N, Rayman G. Inpatient diabetes care during the COVID-19 pandemic: A Diabetes UK rapid review of healthcare professionals' experiences using semi-structured interviews. Diabet Med. 2021 Jan;38(1):e14442. PMID: 33112438. doi: 10.1111/dme.14442.

29. Butler D, Davies-Husband C, Dhanda J, Francis I, Gulati A, Kapoor K, et al. Head and neck oncological ablation and reconstruction in the COVID-19 era - our experience to date. Br J Oral Maxillofac Surg. 2020 Oct;58(8):1008-13. PMID: 32576467. doi: 10.1016/j.bjoms.2020.06.011.

30. Byrne E, Watkinson S. Patient and clinician satisfaction with video consultations during the COVID-19 pandemic: an opportunity for a new way of working. J Orthod. 2021 Mar;48(1):64-73. PMID: 33251951. doi: 10.1177/1465312520973677.

31. Capozzo R, Zoccolella S, Musio M, Barone R, Accogli M, Logroscino G. Telemedicine is a useful tool to deliver care to patients with Amyotrophic Lateral Sclerosis during COVID-19 pandemic: results from Southern Italy. Amyotroph Lateral Scler Frontotemporal Degener. 2020 Nov;21(7-8):542-8. PMID: 32530314. doi: 10.1080/21678421.2020.1773502.

32. Caravatta L, Rosa C, Di Sciascio MB, Tavella Scaringi A, Di Pilla A, Ursini LA, et al. COVID-19 and radiation oncology: the experience of a two-phase plan within a single institution in central Italy. Radiat Oncol. 2020 Sep 29;15(1):226. PMID: 32993690. doi: 10.1186/s13014-020-01670-9.

33. Carter E, Currie CC, Asuni A, Goldsmith R, Toon G, Horridge C, et al. The first six weeks - setting up a UK urgent dental care centre during the COVID-19 pandemic. Br Dent J. 2020 Jun;228(11):842-8. PMID: 32541745. doi: 10.1038/s41415-020-1708-2.

34. Casella D, Fusario D, Cassetti D, Miccoli S, Pesce AL, Bernini A, et al. The patient's pathway for breast cancer in the COVID-19 era: An Italian single-center experience. Breast J. 2020 Aug;26(8):1589-92. PMID: 32596965. doi: 10.1111/tbj.13958.

35. Ceccato F, Voltan G, Sabbadin C, Camozzi V, Merante Boschin I, Mian C, et al. Tele-medicine versus face-to-face consultation in Endocrine Outpatients Clinic during COVID-19 outbreak: a single-center experience during the lockdown period. J Endocrinol Invest. 2021 Aug;44(8):1689-98. PMID: 33355915. doi: 10.1007/s40618-020-01476-2.

36. Cervantes-Torres L, Romero-Blanco C. Longitudinal study of the flash glucose monitoring system in type 1 diabetics: An mHealth ally in times of COVID-19. J Clin Nurs. 2023 Jul;32(13-14):3840-51. PMID: 36071646. doi: 10.1111/jocn.16523.

37. Chana M, Muse S, Ball S, Bennett R, McCarthy R. Critical limb ischaemia in the time of COVID-19: establishing ambulatory service provision. Ann R Coll Surg Engl. 2022 Nov;104(9):673-7. PMID: 34941433. doi: 10.1308/rcsann.2021.0294.

38. Chappell P, Dias A, Bakhai M, Ledger J, Clarke GM. How is primary care access changing? A retrospective, repeated cross-sectional study of patient-initiated demand at general practices in England using a modern access model, 2019-2022. BMJ Open. 2023 Aug 17;13(8):e072944. PMID: 37591638. doi: 10.1136/bmjopen-2023-072944.

39. Chesser TJS, Handley R, Kloos J, De Wachter G, Putzeys G, Gomez-Vallejo J, et al. International trauma care: initial European approaches during the COVID 19 pandemic. OTA Int. 2021 Mar;4(1 Suppl):e112. PMID: 38630066. doi: 10.1097/OI9.0000000000000112.

40. Chu F, Zocchi J, De Berardinis R, Bandi F, Pietrobon G, Scaglione D, et al. COVID-19 and head and neck cancer management. Experience of an oncological hub comprehensive cancer centre and literature review. Acta Otorhinolaryngol Ital. 2022 Apr;42(Suppl. 1):S79-S86. PMID: 35763278. doi: 10.14639/0392-100X-suppl.1-42-2022-09.

41. Chukwusa E, Barclay S, Gulliford M, Harding R, Higginson I, Verne J. General practice service use at the end-of-life before and during the COVID-19 pandemic: a population-based cohort study using primary care electronic health records. BJGP Open. 2024 Apr;8(1). PMID: 37993135. doi: 10.3399/BJGPO.2023.0108.

42. Cohidon C, El Hakmaoui F, Senn N. The role of general practitioners in managing the COVID-19 pandemic in a private healthcare system. Fam Pract. 2022 Jul 19;39(4):586-91. PMID: 34537836. doi: 10.1093/fampra/cmab112.

43. Collins PM, Madden A, O'Connell C, Omer SA, Shakeel Inder M, Casey RG, et al. Urological service provision during the COVID-19 period: the experience from an Irish tertiary centre. Ir J Med Sci. 2021 May;190(2):455-60. PMID: 32856269. doi: 10.1007/s11845-020-02352-x.

44. Corea F, Ciotti S, Cometa A, De Carlo C, Martini G, Baratta S, et al. Telemedicine during the Coronavirus Disease (COVID-19) Pandemic: A Multiple Sclerosis (MS) Outpatients Service Perspective. Neurol Int. 2021 Jan 18;13(1):25-31. PMID: 33477432. doi: 10.3390/neurolint13010003.

45. Crowley D, Delargy I. A national model of remote care for assessing and providing opioid agonist treatment during the COVID-19 pandemic: a report. Harm Reduct J. 2020 Jul 17;17(1):49. PMID: 32680520. doi: 10.1186/s12954-020-00394-z.

46. Crusz SM, Hall PE, Earwicker K, Dexter S, Patel-Walker G, Powles T, et al. Providing an acute oncology service during the COVID-19 pandemic. Clin Med (Lond). 2021 Sep;21(5):e548-e51. PMID: 34385297. doi: 10.7861/clinmed.2020-0693.

47. Cuevas Fernández FJ, Quintana AE, López OTG, Galeote JCG, de León AC, Aguirre-Jaime A. The role of telemedicine in the monitoring and control of patients with hypertension during the COVID−19 pandemic: a multicenter study in primary care. Family Medicine & Primary Care Review. 2024;26(4):438-43. doi: 10.5114/fmpcr.2024.144911.

48. Dambha-Miller H, Hounkpatin HO, Morgan-Harrisskitt J, Stuart B, Fraser SDS, Roderick P. Primary care consultations for respiratory tract symptoms during the COVID-19 pandemic: a cohort study including 70,000 people in South West England. Fam Pract. 2022 May 28;39(3):440-6. PMID: 34632504. doi: 10.1093/fampra/cmab127.

49. De Marchi F, Sarnelli MF, Serioli M, De Marchi I, Zani E, Bottone N, et al. Telehealth approach for amyotrophic lateral sclerosis patients: the experience during COVID-19 pandemic. Acta Neurol Scand. 2021 May;143(5):489-96. PMID: 33185886. doi: 10.1111/ane.13373.

50. Del Hoyo J, Millan M, Garrido-Marin A, Nos P, Barreiro-de Acosta M, Bujanda L, et al. Changes in the management of IBD patients since the onset of COVID-19 pandemic. A path toward the implementation of telemedicine in Spain? Gastroenterol Hepatol. 2022 Nov;45(9):697-705. PMID: 34508808. doi: 10.1016/j.gastrohep.2021.08.006.

51. Deml MJ, Minnema J, Dubois J, Senn O, Streit S, Rachamin Y, et al. The impact of the COVID-19 pandemic on the continuity of care for at-risk patients in Swiss primary care settings: A mixed-methods study. Soc Sci Med. 2022 Apr;298:114858. PMID: 35247784. doi: 10.1016/j.socscimed.2022.114858.

52. Denti M, Pecorari A, Accogli MA, Costi S, Mainini C, Pellegrini M, et al. Facing the COVID-19 pandemic: An Italian feasibility study of a mixed in-person/telerehabilitation intervention for cancer patients. Cancer Med. 2024 Aug;13(15):e70022. PMID: 39095954. doi: 10.1002/cam4.70022.

53. Doica IP, Florescu DN, Oancea CN, Turcu-Stiolica A, Subtirelu MS, Dumitra G, et al. Telemedicine Chronic Viral Hepatitis C Treatment during the Lockdown Period in Romania: A Pilot Study. Int J Environ Res Public Health. 2021 Apr 1;18(7). PMID: 33916226. doi: 10.3390/ijerph18073694.

54. Dunkerley S, Thelwall C, Omiawele J, Smith A, Deo S, Lowdon I. Patient care modifications and hospital regulations during the COVID-19 crisis created inequality and functional hazard for patients with orthopaedic trauma. Int Orthop. 2020 Dec;44(12):2481-5. PMID: 32767088. doi: 10.1007/s00264-020-04764-x.

55. Dzakula A, Banadinovic M, Lovrencic IL, Vajagic M, Dimova A, Rohova M, et al. A comparison of health system responses to COVID-19 in Bulgaria, Croatia and Romania in 2020. Health Policy. 2022 May;126(5):456-64. PMID: 35221121. doi: 10.1016/j.healthpol.2022.02.003.

56. Eger K, Paroczai D, Bacon A, Schleich F, Sergejeva S, Bourdin A, et al. The effect of the COVID-19 pandemic on severe asthma care in Europe: will care change for good? ERJ Open Res. 2022 Apr;8(2). PMID: 35582679. doi: 10.1183/23120541.00065-2022.

57. Eide TB, van Poel E, Willems S, Jacobsen FF. Changes in work tasks and organization of general practice in Norway during the COVID‑19 pandemic: results from a comparative international study. BMC Prim Care. 2023;24(227). doi: 10.1080/13814788.2023.2248374.

58. El Moazen G, Pfeifer B, Loid A, Kastner P, Ciardi C. The Effectiveness of Telemedical Monitoring Program DiabCare Tirol for Patients with Gestational Diabetes Mellitus. Stud Health Technol Inform. 2021 Oct 27;285:205-10. PMID: 34734875. doi: 10.3233/SHTI210599.

59. Faria G, Onubogu IK, Tadros BJ, Relwani J. Change in practice due to COVID-19 - Early experiences of a United Kingdom district general hospital in trauma & orthopaedics. J Orthop. 2020 Nov-Dec;22:288-90. PMID: 32565644. doi: 10.1016/j.jor.2020.06.004.

60. Fieux M, Duret S, Bawazeer N, Denoix L, Zaouche S, Tringali S. Telemedicine for ENT: Effect on quality of care during Covid-19 pandemic. Eur Ann Otorhinolaryngol Head Neck Dis. 2020 Sep;137(4):257-61. PMID: 32624390. doi: 10.1016/j.anorl.2020.06.014.

61. Florea M, Lazea C, Gaga R, Sur G, Lotrean L, Puia A, et al. Lights and Shadows of the Perception of the Use of Telemedicine by Romanian Family Doctors During the COVID-19 Pandemic. Int J Gen Med. 2021;14:1575-87. PMID: 33953605. doi: 10.2147/IJGM.S309519.

62. Fonseca M, MacKenna B, Mehrkar A, Open SC, Walters CE, Hickman G, et al. The Use of Online Consultation Systems or Remote Consulting in England Characterized Through the Primary Care Health Records of 53 Million People in the OpenSAFELY Platform: Retrospective Cohort Study. JMIR Public Health Surveill. 2024 Sep 18;10:e46485. PMID: 39292500. doi: 10.2196/46485.

63. Forrester M, Breitenfeld L, Castelo-Branco M, Aperta J. The Effects of the COVID-19 Pandemic in Oncology Patient Management. Int J Environ Res Public Health. 2022 Jul 25;19(15). PMID: 35897414. doi: 10.3390/ijerph19159041.

64. Fuentes B, Alonso de Lecinana M, Calleja-Castano P, Carneado-Ruiz J, Egido-Herrero J, Gil-Nunez A, et al. [Impact of the COVID-19 pandemic on the organisation of stroke care. Madrid Stroke Care Plan]. Neurologia (Engl Ed). 2020 Jul-Aug;35(6):363-71. PMID: 32563566. doi: 10.1016/j.nrl.2020.05.007.

65. Gabriel C, Mathiot A, Boumediane M, Vernet P, Schwebel M, de Figueiredo C, et al. Organization of outpatient consultations at a hand surgery department in a French university hospital during the COVID-19 lockdown. Hand Surg Rehabil. 2021 Feb;40(1):17-24. PMID: 33130022. doi: 10.1016/j.hansur.2020.10.006.

66. Garrido-Cumbrera M, Marzo-Ortega H, Christen L, Plazuelo-Ramos P, Webb D, Jacklin C, et al. Assessment of impact of the COVID-19 pandemic from the perspective of patients with rheumatic and musculoskeletal diseases in Europe: results from the REUMAVID study (phase 1). RMD Open. 2021;7. doi: 10.1136/rmdopen-2020-001546.

67. Gebbia V, Piazza D, Valerio MR, Borsellino N, Firenze A. Patients With Cancer and COVID-19: A WhatsApp Messenger-Based Survey of Patients' Queries, Needs, Fears, and Actions Taken. JCO Glob Oncol. 2020 May;6:722-9. PMID: 32412811. doi: 10.1200/GO.20.00118.

68. Geerdink TH, Salentijn DA, de Vries KA, Noordman PCW, van Dongen JM, Haverlag R, et al. Optimizing orthopedic trauma care delivery during the COVID-19 pandemic. A closed-loop audit of implementing a virtual fracture clinic and fast-track pathway in a Dutch level 2 trauma center. Trauma Surg Acute Care Open. 2021;6(1):e000691. PMID: 34632079. doi: 10.1136/tsaco-2021-000691.

69. Gesuete FP, Molle M, Gubitosi A, Izzo S, Todde S, Nicoletti GF, et al. Telemonitoring Wound Recovery with Smartphone: An Italian Experience during Pandemic Period. Plast Reconstr Surg Glob Open. 2023 May;11(5):e5076. PMID: 37250826. doi: 10.1097/GOX.0000000000005076.

70. Gilbert AW, Billany JCT, Adam R, Martin L, Tobin R, Bagdai S, et al. Rapid implementation of virtual clinics due to COVID-19: report and early evaluation of a quality improvement initiative. BMJ Open Qual. 2020 May;9(2). PMID: 32439740. doi: 10.1136/bmjoq-2020-000985.

71. Giudice A, Barone S, Muraca D, Averta F, Diodati F, Antonelli A, et al. Can Teledentistry Improve the Monitoring of Patients during the Covid-19 Dissemination? A Descriptive Pilot Study. Int J Environ Res Public Health. 2020 May 13;17(10). PMID: 32414126. doi: 10.3390/ijerph17103399.

72. Gleeson LL, Ludlow A, Wallace E, Argent R, Collins C, Clyne B, et al. Changes to primary care delivery during the COVID-19 pandemic and perceived impact on medication safety: A survey study. Explor Res Clin Soc Pharm. 2022 Jun;6:100143. PMID: 35702683. doi: 10.1016/j.rcsop.2022.100143.

73. Gomes-de Almeida S, Marabujo T, do Carmo-Gonçalves M. [Telemedicine satisfaction of primary care patients during COVID-19 pandemics]. Medicina de Familia SEMERGEN 2021 May-Jun;47(4):248-55. PMID: 33781673. doi: 10.1016/j.semerg.2021.01.005.

74. Gonzalez HA, Myers S, Whitehead E, Pattinson A, Stamp K, Turnbull J, et al. React, reset and restore: Adaptation of a large inflammatory bowel disease service during COVID-19 pandemic. Clin Med (Lond). 2020 Sep;20(5):e183-e8. PMID: 32719036. doi: 10.7861/clinmed.2020-0369.

75. Grobe-Einsler M, Taheri Amin A, Faber J, Schaprian T, Jacobi H, Schmitz-Hubsch T, et al. Development of SARA(home) , a New Video-Based Tool for the Assessment of Ataxia at Home. Mov Disord. 2021 May;36(5):1242-6. PMID: 33433030. doi: 10.1002/mds.28478.

76. Hardman JC, Tikka T, Paleri V, Ent Uk B, Integrate. Remote triage incorporating symptom-based risk stratification for suspected head and neck cancer referrals: A prospective population-based study. Cancer. 2021 Nov 15;127(22):4177-89. PMID: 34411287. doi: 10.1002/cncr.33800.

77. Hartl L, Semmler G, Hofer BS, Schirwani N, Jachs M, Simbrunner B, et al. COVID-19-Related Downscaling of In-Hospital Liver Care Decreased Patient Satisfaction and Increased Liver-Related Mortality. Hepatol Commun. 2021 Oct;5(10):1660-75. PMID: 34222742. doi: 10.1002/hep4.1758.

78. Indini A, Aschele C, Cavanna L, Clerico M, Daniele B, Fiorentini G, et al. Reorganisation of medical oncology departments during the novel coronavirus disease-19 pandemic: a nationwide Italian survey. Eur J Cancer. 2020 Jun;132:17-23. PMID: 32311643. doi: 10.1016/j.ejca.2020.03.024.

79. Jacome C, Pereira AM, Amaral R, Alves-Correia M, Almeida R, Mendes S, et al. The use of remote care during the Coronavirus disease 2019 pandemic: a perspective of Portuguese and Spanish physicians. Eur Ann Allergy Clin Immunol. 2022 Jan;54(1):25-9. PMID: 33354962. doi: 10.23822/EurAnnACI.1764-1489.184.

80. Johansson JK, Korhonen P. Utilisation of primary healthcare services by patients with hypertension before, during and after the COVID-19 pandemic in Turku, Finland-are digital services creating disparity? Scand J Public Health. 2025 Nov 12:14034948251392076. PMID: 41229105. doi: 10.1177/14034948251392076.

81. Johnsen TM, Norberg BL, Kristiansen E, Zanaboni P, Austad B, Krogh FH, et al. Suitability of Video Consultations During the COVID-19 Pandemic Lockdown: Cross-sectional Survey Among Norwegian General Practitioners. J Med Internet Res. 2021 Feb 8;23(2):e26433. PMID: 33465037. doi: 10.2196/26433.

82. Joy M, McGagh D, Jones N, Liyanage H, Sherlock J, Parimalanathan V, et al. Reorganisation of primary care for older adults during COVID-19: a cross-sectional database study in the UK. Br J Gen Pract. 2020 Aug;70(697):e540-e7. PMID: 32661009. doi: 10.3399/bjgp20X710933.

83. Kaddour H, Jama GM, Stagnell S, Kaddour S, Guner K, Kumar G. Remote triaging of urgent suspected head and neck cancer referrals: our experience during the first wave of the COVID-19 pandemic. Eur Arch Otorhinolaryngol. 2022 Feb;279(2):1111-5. PMID: 34661717. doi: 10.1007/s00405-021-07135-3.

84. Kenis I, Theys S, Hermie E, Foulon V, Van Hecke A. Impact of COVID-19 on the Organization of Cancer Care in Belgium: Lessons Learned for the (Post-)Pandemic Future. Int J Environ Res Public Health. 2022 Sep 30;19(19). PMID: 36231756. doi: 10.3390/ijerph191912456.

85. Kilduff CL, Thomas AA, Dugdill J, Casswell EJ, Dabrowski M, Lovegrove C, et al. Creating the Moorfields' virtual eye casualty: video consultations to provide emergency teleophthalmology care during and beyond the COVID-19 pandemic. BMJ Health Care Inform. 2020 Aug;27(3). PMID: 32796085. doi: 10.1136/bmjhci-2020-100179.

86. King R, Carolan C, Robertson S. COVID-19: a catalyst for change in remote and rural advanced clinical practice – A qualitative study. Journal of Integrated Care. 2023;32(1):63-73. doi: 10.1108/jica-03-2023-0014.

87. Kirk UB, Hostrup Vestergaard C, Hammer Bech B, Bondo Christensen M, Kallestrup P, Huibers L. Video consultation in general practice during COVID-19: a register-based study in Denmark. BJGP Open. 2024 Jul;8(2). PMID: 38191187. doi: 10.3399/BJGPO.2023.0208.

88. Klain M, Nappi C, Maurea S, De Risi M, Volpe F, Caiazzo E, et al. Management of differentiated thyroid cancer through nuclear medicine facilities during Covid-19 emergency: the telemedicine challenge. Eur J Nucl Med Mol Imaging. 2021 Mar;48(3):831-6. PMID: 32965559. doi: 10.1007/s00259-020-05041-0.

89. Kollmann J, Sana S, Magnee T, Boer S, Merkelbach I, Kocken PL, et al. Patients' and professionals' experiences with remote care during COVID-19: a qualitative study in general practices in low-income neighborhoods. Prim Health Care Res Dev. 2024 Jun 3;25:e32. PMID: 38826073. doi: 10.1017/S1463423624000240.

90. Krawczyk A, Marszalek M. A Comparative Analysis of Telerehabilitation and Telemedicine Utilization During the COVID-19 Pandemic in Poland: Trends, Patterns, and Implications. International Journal of Telerehabilitation. 2024;16(1). doi: 10.5195/ijt.2024.6627.

91. Lapão LV, Peyroteo M, Maia M, Seixas J, Gregório J, Mira da Silva M, et al. Implementation of Digital Monitoring Services During the COVID-19 Pandemic for Patients With Chronic Diseases: Design Science Approach. J Med Internet Res. 2021 Aug 26;23(8):e24181. PMID: 34313591. doi: 10.2196/24181.

92. Legrottaglie EF, Balia L, Camesasca FI, Vallejo-Garcia JL, Fossati G, Vinciguerra R, et al. Management of an ophthalmology department during COVID-19 pandemic in Milan, Italy. Eur J Ophthalmol. 2021 Sep;31(5):2259-67. PMID: 32962415. doi: 10.1177/1120672120960334.

93. Li JO, Thomas AAP, Kilduff CLS, Logeswaran A, Ramessur R, Jaselsky A, et al. Safety of video-based telemedicine compared to in-person triage in emergency ophthalmology during COVID-19. EClinicalMedicine. 2021 Apr;34:100818. PMID: 33842860. doi: 10.1016/j.eclinm.2021.100818.

94. Loezar-Hernandez M, Briones-Vozmediano E, Gea-Sanchez M, Robledo-Martin J, Otero-Garcia L. Primary health care midwives' perceptions on the use of telemedicine during the COVID-19 pandemic in Spain. Enferm Clin (Engl Ed). 2023 Nov-Dec;33(6):380-90. PMID: 37898171. doi: 10.1016/j.enfcle.2023.10.002.

95. Luciani LG, Mattevi D, Cai T, Giusti G, Proietti S, Malossini G. Teleurology in the Time of Covid-19 Pandemic: Here to Stay? Urology. 2020 Jun;140:4-6. PMID: 32298686. doi: 10.1016/j.urology.2020.04.004.

96. Luengo-Alonso G, García-Seisdedos Pérez-Tabernero F, Tovar-Bazaga M, Arguello-Cuenca JM, Calvo E. Critical adjustments in a department of orthopaedics through the COVID-19 pandemic. Int Orthop. 2020 Aug;44(8):1557-64. PMID: 32474718. doi: 10.1007/s00264-020-04647-1.

97. Mackintosh L, Busby A, Farrington K, Hawkins J, Afuwape S, Bristow P, et al. Impact of the COVID-19 pandemic on services for patients with chronic kidney disease: findings of a national survey of UK kidney centres. BMC Nephrol. 2023 Dec 4;24(1):356. PMID: 38049710. doi: 10.1186/s12882-023-03344-6.

98. Manzi G, Papa S, Mariani MV, Scoccia G, Filomena D, Malerba C, et al. Telehealth: A winning weapon to face the COVID-19 outbreak for patients with pulmonary arterial hypertension. Vascul Pharmacol. 2022 Aug;145:107024. PMID: 35716991. doi: 10.1016/j.vph.2022.107024.

99. Mathai NJ, Venkatesan AS, Key T, Wilson C, Mohanty K. COVID-19 and orthopaedic surgery: evolving strategies and early experience. Bone Jt Open. 2020 May;1(5):160-6. PMID: 33241227. doi: 10.1302/2633-1462.15.BJO-2020-0021.R1.

100. Mazzone P, Peretto G, Radinovic A, Limite LR, Marzi A, Sala S, et al. The COVID-19 challenge to cardiac electrophysiologists: optimizing resources at a referral center. J Interv Card Electrophysiol. 2020;59(2):321-7. PMID: 32425656. doi: 10.1007/s10840-020-00761-7.

101. McKenna MC, Al-Hinai M, Bradley D, Doran E, Hunt I, Hutchinson S, et al. Patients' Experiences of Remote Neurology Consultations during the COVID-19 Pandemic. Eur Neurol. 2020;83(6):622-5. PMID: 33147591. doi: 10.1159/000511900.

102. Miceli L, Dal Mas F, Biancuzzi H, Bednarova R, Rizzardo A, Cobianchi L, et al. Doctor@Home: Through a Telemedicine Co-production and Co-learning Journey. J Cancer Educ. 2022 Aug;37(4):1236-8. PMID: 33442862. doi: 10.1007/s13187-020-01945-5.

103. Miguela Álvarez SM, Bartra Ylla A, Salvador Carreño J, Castillón P, García Cardona C, Anglès Crespo F. Telephone consultation service in orthopedics during COVID-19 pandemic. Rev Esp Cir Ortop Traumatol (Engl Ed). 2021 May-Jun;65(3):167-71. PMID: 34040680. doi: 10.1016/j.recote.2020.07.007.

104. Minghelli B, Soares A, Guerreiro A, Ribeiro A, Cabrita C, Vitoria C, et al. Physiotherapy services in the face of a pandemic. Rev Assoc Med Bras (1992). 2020 Apr;66(4):491-7. PMID: 32578784. doi: 10.1590/1806-9282.66.4.491.

105. Minniti A, Maglione W, Pignataro F, Cappadona C, Caporali R, Del Papa N. Taking care of systemic sclerosis patients during COVID-19 pandemic: rethink the clinical activity. Clin Rheumatol. 2020 Jul;39(7):2063-5. PMID: 32462423. doi: 10.1007/s10067-020-05191-4.

106. Montesi G, Di Biase S, Chierchini S, Pavanato G, Virdis GE, Contato E, et al. Radiotherapy during COVID-19 pandemic. How to create a No fly zone: a Northern Italy experience. Radiol Med. 2020 Jun;125(6):600-3. PMID: 32415473. doi: 10.1007/s11547-020-01217-8.

107. Morgan C, Ahluwalia AK, Aframian A, Li L, Sun SNM. The impact of the novel coronavirus on trauma and orthopaedics in the UK. Br J Hosp Med (Lond). 2020 Apr 2;81(4):1-6. PMID: 32343187. doi: 10.12968/hmed.2020.0137.

108. Morreel S, Philips H, Verhoeven V. Organisation and characteristics of out-of-hours primary care during a COVID-19 outbreak: A real-time observational study. PLoS One. 2020;15(8):e0237629. PMID: 32790804. doi: 10.1371/journal.pone.0237629.

109. Motolese F, Magliozzi A, Puttini F, Rossi M, Capone F, Karlinski K, et al. Parkinson's Disease Remote Patient Monitoring During the COVID-19 Lockdown. Front Neurol. 2020;11:567413. PMID: 33117262. doi: 10.3389/fneur.2020.567413.

110. Mugnai G, Volpiana A, Cavedon S, Paolini C, Perrone C, Bilato C. Boosting telemedicine through remote monitoring of cardiac electronic devices during the Italian COVID-19 outbreak. Cardiol J. 2021;28(2):336-8. PMID: 33634839. doi: 10.5603/CJ.a2021.0011.

111. Munda A, Indihar BS, Okanovic G, Zorko K, Steblovnik L, Barlovic DP. Maternal and Perinatal Outcomes During the COVID-19 Epidemic in Pregnancies Complicated by Gestational Diabetes. Zdr Varst. 2023 Mar;62(1):22-9. PMID: 36694793. doi: 10.2478/sjph-2023-0004.

112. Murphy M, Scott LJ, Salisbury C, Turner A, Scott A, Denholm R, et al. Implementation of remote consulting in UK primary care following the COVID-19 pandemic: a mixed-methods longitudinal study. Br J Gen Pract. 2021;71(704):e166-e77. PMID: 33558332. doi: 10.3399/BJGP.2020.0948.

113. Murris F, Huchon C, Zilberman S, Dabi Y, Phalippou J, Canlorbe G, et al. Impact of the first lockdown for coronavirus 19 on breast cancer management in France: A multicentre survey. J Gynecol Obstet Hum Reprod. 2021 Nov;50(9):102166. PMID: 34033966. doi: 10.1016/j.jogoh.2021.102166.

114. Muschol J, Heinrich M, Heiss C, Hernandez AM, Knapp G, Repp H, et al. Digitization of Follow-Up Care in Orthopedic and Trauma Surgery With Video Consultations: Health Economic Evaluation Study From a Health Provider's Perspective. J Med Internet Res. 2023 Dec 25;25:e46714. PMID: 38145481. doi: 10.2196/46714.

115. Muthiah S, Craig FE, Sinclair S, Wylie G, Torley D, Wong TH, et al. Rapid Expansion of a Teledermatology Web Application for Digital Dermatology Assessment Necessitated by the COVID-19 Pandemic: Retrospective Evaluation. JMIR Dermatol. 2023 Jul 26;6:e36307. PMID: 37632929. doi: 10.2196/36307.

116. Nebsbjerg MA, Vestergaard CH, Bomholt KB, Christensen MB, Huibers L. Use of Video in Telephone Triage in Out-of-Hours Primary Care: Register-Based Study. JMIR Med Inform. 2024 Apr 4;12:e47039. PMID: 38596835. doi: 10.2196/47039.

117. Newby K, Howlett N, Wagner AP, Smeeton N, Fakoya O, Lloyd N, et al. Moving an exercise referral scheme to remote delivery during the Covid-19 pandemic: an observational study examining the impact on uptake, adherence, and costs. BMC Public Health. 2024 Aug 27;24(1):2324. PMID: 39192229. doi: 10.1186/s12889-024-19392-y.

118. Nielsen JB, Andersen HS. Investigating changes in user and diagnostic patterns in general practice during the COVID pandemic in 2020: a cohort study using Danish patient data from two consecutive years before and during the pandemic. BMJ Open. 2025 Jun 4;15(6):e096243. PMID: 40467322. doi: 10.1136/bmjopen-2024-096243.

119. Noé E, Navarro MD, Amorés D, García-Blázquez MC, O'Valle M, Villarino P, et al. [Effectiveness, adherence and usability of a teleneurorehabilitation programme to ensure continuity of care for patients with acquired brain injury during the COVID-19 pandemic]. Rev Neurol. 2021 Nov 16;73(10):345-50. PMID: 34755887. doi: 10.33588/rn.7310.2021275.

120. Nune A, Iyengar K, Ahmed A, Sapkota H. Challenges in delivering rheumatology care during COVID-19 pandemic. Clin Rheumatol. 2020 Sep;39(9):2817-21. PMID: 32712743. doi: 10.1007/s10067-020-05312-z.

121. Nuñez JH, Porcel JA, Pijoan J, Batalla L, Teixidor J, Guerra-Farfan E, et al. Rethinking Trauma Hospital Services in one of Spain's Largest University Hospitals during the COVID-19 pandemic. How can we organize and help? Our experience. Injury. 2020 Dec;51(12):2827-33. PMID: 33004206. doi: 10.1016/j.injury.2020.09.055.

122. O'Donovan M, Buckley C, Benson J, Roche S, McGowan M, Parkinson L, et al. Telehealth for delivery of haemophilia comprehensive care during the COVID-19 pandemic. Haemophilia. 2020 Nov;26(6):984-90. PMID: 32997849. doi: 10.1111/hae.14156.

123. Ochieng L, Salehi M, Ochieng R, Nijhof D, Wong R, Gupta V, et al. Augmented video consultations in care homes during the COVID-19 pandemic: a qualitative study. BJGP Open. 2022 Dec;6(4). PMID: 35764408. doi: 10.3399/BJGPO.2022.0073.

124. Ohnleiter T, Piot L, Rogenmuser A, Noirclerc M, Hamlaoui R, Grandgirard A. [Management of a radiotherapy center during the COVID-19 outbreak: The experience of the Mulhouse hospital centre (France)]. Cancer Radiother. 2020 Jun;24(3):188-93. PMID: 32334905. doi: 10.1016/j.canrad.2020.04.002.

125. Omboni SaB, T. and Rizzi, F. and Tomassini, F. and Panzeri, E. and Campolo, L. Telehealth at scale can improve chronic disease management in the community during a pandemic: An experience at the time of COVID-19. PLoS One. 2021;16(9). doi: https://doi.org/10.1371/journal.pone.0258015.

126. Paleri V, Hardman J, Tikka T, Bradley P, Pracy P, Kerawala C. Rapid implementation of an evidence-based remote triaging system for assessment of suspected referrals and patients with head and neck cancer on follow-up after treatment during the COVID-19 pandemic: Model for international collaboration. Head Neck. 2020 Jul;42(7):1674-80. PMID: 32374942. doi: 10.1002/hed.26219.

127. Papoutsi C, Shaw S, Greenhalgh T. Implementing video group consultations in general practice during COVID-19: a qualitative study. Br J Gen Pract. 2022 Jul;72(720):e483-e91. PMID: 35636969. doi: 10.3399/BJGP.2021.0673.

128. Pareyson D, Pantaleoni C, Eleopra R, De Filippis G, Moroni I, Freri E, et al. Neuro-telehealth for fragile patients in a tertiary referral neurological institute during the COVID-19 pandemic in Milan, Lombardy. Neurol Sci. 2021 Jul;42(7):2637-44. PMID: 33929645. doi: 10.1007/s10072-021-05252-9.

129. Pascual J, Mazuecos A, Sanchez-Antolin G, Sole A, Ventura-Aguiar P, Crespo M, et al. Best practices during COVID-19 pandemic in solid organ transplant programs in Spain. Transplant Rev (Orlando). 2023 Jan;37(1):100749. PMID: 36889117. doi: 10.1016/j.trre.2023.100749.

130. Paudyal VC, C.; Fialová, D.; Henman, M. C.; Hazen, A.; Okuyan, B.; Lutters, M.; Stewart, D. Provision of clinical pharmacy services during the COVID-19 pandemic: Experiences of pharmacists from 16 European countries. Research in Social and Administrative Pharmacy. 2021;17:1507–17.

131. Peretto GDL, G.; Campochiaro, C.; Palmisano, A.; Busnardo, E.; Sartorelli, S.; Barzaghi, F.; Cicalese, M.; Esposito, A.; Sala, S. Telemedicine in myocarditis: Evolution of a mutidisciplinary “disease unit” at the time of COVID-19 pandemic. Am Heart J. 2020;229:121-6.

132. Perrin A, Mainard N, Limousin M, Meyer E, Remy F, Strouk G, et al. Satisfaction and feasibility of videoconsultation (VC) in orthopaedic and trauma surgery in the context of the COVID-19 pandemic: Prospective study of 783 patients. Orthop Traumatol Surg Res. 2023 Feb;109(1):103345. PMID: 35671985. doi: 10.1016/j.otsr.2022.103345.

133. Perrone AM, Dondi G, Giunchi S, De Crescenzo E, Boussedra S, Tesei M, et al. COVID-19 free oncologic surgical hub: The experience of reallocation of a gynecologic oncology unit during pandemic outbreak. Gynecol Oncol. 2021 Apr;161(1):89-96. PMID: 33223219. doi: 10.1016/j.ygyno.2020.09.030.

134. Phillips GSA, Wormald JCR, Yoshimura R, Gardiner MD, Rodrigues JN, Collins DP, et al. RSTNCOVID Burns: A multi-centre service evaluation and stakeholder survey of the impact of COVID-19 on burns care in England, Wales and Northern Ireland. J Plast Reconstr Aesthet Surg. 2022 May;75(5):1602-9. PMID: 34955398. doi: 10.1016/j.bjps.2021.11.086.

135. Piane M, Bianco L, Mancini R, Fornelli P, Gabriele A, Medici F, et al. Impact of the COVID-19 Pandemic on Clinical Pathways for Non-SARS-CoV-2 Related Diseases in the Lazio Region, Italy. Int J Environ Res Public Health. 2022 Jan 6;19(2). PMID: 35055455. doi: 10.3390/ijerph19020635.

136. Pignatti M, Pinto V, Miralles MEL, Giorgini FA, Cannamela G, Cipriani R. How the COVID-19 pandemic changed the Plastic Surgery activity in a regional referral center in Northern Italy. J Plast Reconstr Aesthet Surg. 2020 Jul;73(7):1348-56. PMID: 32499187. doi: 10.1016/j.bjps.2020.05.002.

137. Pinar U, Anract J, Perrot O, Tabourin T, Chartier-Kastler E, Parra J, et al. Preliminary assessment of patient and physician satisfaction with the use of teleconsultation in urology during the COVID-19 pandemic. World J Urol. 2021 Jun;39(6):1991-6. PMID: 32909174. doi: 10.1007/s00345-020-03432-4.

138. Piro A, Magnocavallo M, Della Rocca DG, Neccia M, Manzi G, Mariani MV, et al. Management of cardiac implantable electronic device follow-up in COVID-19 pandemic: Lessons learned during Italian lockdown. J Cardiovasc Electrophysiol. 2020 Nov;31(11):2814-23. PMID: 32954600. doi: 10.1111/jce.14755.

139. Pulvirenti F, Cinetto F, Milito C, Bonanni L, Pesce AM, Leodori G, et al. Health-Related Quality of Life in Common Variable Immunodeficiency Italian Patients Switched to Remote Assistance During the COVID-19 Pandemic. J Allergy Clin Immunol Pract. 2020 Jun;8(6):1894-9 e2. PMID: 32278865. doi: 10.1016/j.jaip.2020.04.003.

140. Quinn LM, Olajide O, Green M, Sayed H, Ansar H. Patient and Professional Experiences With Virtual Antenatal Clinics During the COVID-19 Pandemic in a UK Tertiary Obstetric Hospital: Questionnaire Study. J Med Internet Res. 2021 Aug 31;23(8):e25549. PMID: 34254940. doi: 10.2196/25549.

141. Rajasekaran RB, Whitwell D, Cosker TDA, Gibbons C. Service delivery during the COVID-19 pandemic: Experience from The Oxford Bone Tumour and Soft Tissue Sarcoma service. J Clin Orthop Trauma. 2020 Jul;11(Suppl 4):S419-S22. PMID: 32774004. doi: 10.1016/j.jcot.2020.05.035.

142. Ramaswami U, D'Amore S, Finnegan N, Hughes D, Kazemi M, Lysosomal Disorders Team RFLNHSFT. Impact of SARS-CoV-2 (COVID-19) pandemic on patients with lysosomal storage disorders and restoration of services: experience from a specialist centre. Intern Med J. 2021 Oct;51(10):1580-93. PMID: 34487419. doi: 10.1111/imj.15473.

143. Rayo MN, Fernandez-Buhigas I, Ferrer E, Arrebola M, Gil MM, Santacruz B. Application of a new protocol for providing obstetric care in an outpatient service during the COVID-19 pandemic in a public hospital in Madrid, Spain. Front Med (Lausanne). 2022;9:902640. PMID: 35991653. doi: 10.3389/fmed.2022.902640.

144. Riboli-Sasco E, El-Osta A, El Asmar ML, Karki M, Kerr G, Sathaymoorthy G, et al. Investigating barriers & facilitators for the successful implementation of the BP@home initiative in London: Primary care perspectives. PLoS One. 2024;19(2):e0298898. PMID: 38422101. doi: 10.1371/journal.pone.0298898.

145. Rimmer MP, Al Wattar BH, Members U. Provision of obstetrics and gynaecology services during the COVID-19 pandemic: a survey of junior doctors in the UK National Health Service. BJOG. 2020 Aug;127(9):1123-8. PMID: 32460422. doi: 10.1111/1471-0528.16313.

146. Rodler S, Apfelbeck M, Schulz GB, Ivanova T, Buchner A, Staehler M, et al. Telehealth in Uro-oncology Beyond the Pandemic: Toll or Lifesaver? Eur Urol Focus. 2020 Sep 15;6(5):1097-103. PMID: 32534969. doi: 10.1016/j.euf.2020.05.010.

147. Romano M, Negrini A, Negrini S. Lessons learned in two months of exclusive application of telephysiotherapy instead of classical physiotherapy during the lockdown in Italy. Spine J. 2021 Mar;21(3):366-9. PMID: 33589094. doi: 10.1016/j.spinee.2020.10.023.

148. Rossi B, Zoccali C, Baldi J, Scotto di Uccio A, Biagini R, De Luca A, et al. Reorganization Tips from a Sarcoma Unit at Time of the COVID-19 Pandemic in Italy: Early Experience from a Regional Referral Oncologic Center. J Clin Med. 2020 Jun 15;9(6). PMID: 32549298. doi: 10.3390/jcm9061868.

149. Runfola MF, G.; Pintus, S.; Iafrancesco, M.; Moroni, R. Telemedicine Implementation on a Bariatric Outpatient Clinic During COVID-19 Pandemic in Italy: an Unexpected Hill-Start. Obesity Surgery. 2020;30:5145–9.

150. Russo V, Cassini R, Caso V, Donno C, Laezza A, Naddei M, et al. Nursing Teleconsultation for the Outpatient Management of Patients with Cardiovascular Disease during COVID-19 Pandemic. Int J Environ Res Public Health. 2021 Feb 21;18(4). PMID: 33669951. doi: 10.3390/ijerph18042087.

151. Rzewuska Diaz M, Locock L, Keen A, Melvin M, Myhill A, Ramsay C. Implementation of a Web-Based Outpatient Asynchronous Consultation Service: Mixed Methods Study. J Med Internet Res. 2024 Jun 4;26:e48092. PMID: 38833695. doi: 10.2196/48092.

152. Sacchi CA, K.; Roczniewska, M.; Luckhaus, J. L.; Malmqvist, M.; Rodmalm, L. P.; Lodin, K.; Mosson, R.; Danapfel, P.; Wannheden, C.; Mazzocato, P. Mind the gap: analysis of two pilot projects of a home telehealth service for persons with complex conditions in a Swedish hospital. BMC Health Services Research. 2023;23(463).

153. Saibeni S, Scucchi L, Dragoni G, Bezzio C, Miranda A, Ribaldone DG, et al. Activities related to inflammatory bowel disease management during and after the coronavirus disease 2019 lockdown in Italy: How to maintain standards of care. United European Gastroenterol J. 2020 Dec;8(10):1228-35. PMID: 33070758. doi: 10.1177/2050640620964132.

154. Saint-Lary O, Gautier S, Le Breton J, Gilberg S, Frappe P, Schuers M, et al. How GPs adapted their practices and organisations at the beginning of COVID-19 outbreak: a French national observational survey. BMJ Open. 2020 Dec 2;10(12):e042119. PMID: 33268433. doi: 10.1136/bmjopen-2020-042119.

155. Salzano A, D'Assante R, Stagnaro FM, Valente V, Crisci G, Giardino F, et al. Heart failure management during the COVID-19 outbreak in Italy: a telemedicine experience from a heart failure university tertiary referral centre. Eur J Heart Fail. 2020 Jun;22(6):1048-50. PMID: 32463534. doi: 10.1002/ejhf.1911.

156. Sanders MW, Field J, Newell D, Osborne N. Use of Remote Consultations by Chiropractors in the United Kingdom During the COVID-19 Pandemic: A Cross-Sectional Survey. Journal of Chiropractic Medicine. 2025;24(1-4):106-18. doi: 10.1016/j.jcm.2025.07.003.

157. Scaldaferri F, Pugliese D, Privitera G, Onali S, Lopetuso LR, Rizzatti G, et al. Impact of COVID-19 pandemic on the daily management of biotechnological therapy in inflammatory bowel disease patients: Reorganisational response in a high-volume Italian inflammatory bowel disease centre. United European Gastroenterol J. 2020 Aug;8(7):775-81. PMID: 32438878. doi: 10.1177/2050640620929133.

158. Schnoor K, Versluis A, Chavannes NH, Talboom-Kamp E. The Usability of Homelab, a Digital Self-service at a Dutch General Practice, for Diagnostic Tests: Pilot Study With a Questionnaire. JMIR Form Res. 2023 Jan 26;7:e42151. PMID: 36701183. doi: 10.2196/42151.

159. Severino P, D'Amato A, Prosperi S, Magnocavallo M, Maraone A, Notari C, et al. Clinical Support through Telemedicine in Heart Failure Outpatients during the COVID-19 Pandemic Period: Results of a 12-Months Follow Up. J Clin Med. 2022 May 16;11(10). PMID: 35628916. doi: 10.3390/jcm11102790.

160. Shah A, Bryant C, Patel J, Tagar H, Akintola D, Obisesan O. COVID-19: establishing an oral surgery-led urgent dental care hub. Br Dent J. 2020 Jun;228(12):957-63. PMID: 32591712. doi: 10.1038/s41415-020-1713-5.

161. Shaw SE, Hughes G, Wherton J, Moore L, Rosen R, Papoutsi C, et al. Achieving Spread, Scale Up and Sustainability of Video Consulting Services During the COVID-19 Pandemic? Findings From a Comparative Case Study of Policy Implementation in England, Wales, Scotland and Northern Ireland. Front Digit Health. 2021;3:754319. PMID: 34988546. doi: 10.3389/fdgth.2021.754319.

162. Sheil OM, F. M. Reorganisation of obstetric services during the COVID pandemic - Experience from National Maternity Hospital Dublin Ireland. Best Practice & Research Clinical Obstetrics and Gynaecology. 2021;73.

163. Silsand L, Severinsen GH, Berntsen G. Preservation of Person-Centered Care Through Videoconferencing for Patient Follow-up During the COVID-19 Pandemic: Case Study of a Multidisciplinary Care Team. JMIR Form Res. 2021 Mar 5;5(3):e25220. PMID: 33646965. doi: 10.2196/25220.

164. Smrke AY, E.; Wilson, R.; Husson, O.; Farag, S.; Merry, E.; Macklin-Doherty, A.; Cojocaru, E.; Arthur, A.; Miah, A. B.; Benson, C.; Zaidi, S.; Gennatas, S.; Jones, R. L. Telemedicine During the COVID-19 Pandemic: Impact on Care for Rare Cancers. JCO Global Oncol. 2020;6:1046-51.

165. Solans O, Vidal-Alaball J, Roig Cabo P, Mora N, Coma E, Bonet Simó JM, et al. Characteristics of Citizens and Their Use of Teleconsultations in Primary Care in the Catalan Public Health System Before and During the COVID-19 Pandemic: Retrospective Descriptive Cross-sectional Study. J Med Internet Res. 2021 May 27;23(5):e28629. PMID: 33970867. doi: 10.2196/28629.

166. Solberg Carlsson K, Ovretveit J, Ohrling M. Rapid implementation of remote digital primary care in Stockholm and implications for further system-wide implementation: practitioner's and manager's experience of the Always Open mobile application. Scand J Prim Health Care. 2023 Sep;41(3):232-46. PMID: 37470469. doi: 10.1080/02813432.2023.2229387.

167. Soler MJMH, M.; Ortiz, A.; del Pino y Pino, M. D.; Salgueira Lazo, M. Impacto de la pandemia COVID-19 en los servicios de Nefrologia espanoles. Nefrologia. 2020;40(6):579–84.

168. Somani BKP, A.; Coulter, P.; Smith, J. Delivery of urological services (telemedicine and urgent surgery) during COVID-19 lockdown: experience and lessons learnt from a university hospital in United Kingdom. Scottish Medical Journal. 2020;0(0):1-3.

169. Stansfield JD, S.; Harrison, R.; Lee, K.; Sharma, S.; Okour, K.; Agrawal, S.; Miah, M. S. Management of ENT emergencies during the coronavirus disease 2019 pandemic. J Laryngol Otol. 2021:1-8.

170. Stepaniuk A, Pawlukianiec C, Krawiel M, Lewoc M, Baran A, Flisiak I. Great hopes or disappointment - a survey-based study on patients' and doctors' perception of telemedicine during the COVID-19 pandemic in Poland. Postepy Dermatol Alergol. 2022 Apr;39(2):384-91. PMID: 35645687. doi: 10.5114/ada.2022.113827.

171. Stewart J, McCorry N, Reid H, Hart N, Kee F. Implementation of remote asthma consulting in general practice in response to the COVID-19 pandemic: an evaluation using extended Normalisation Process Theory. BJGP Open. 2022 Mar;6(1). PMID: 34862167. doi: 10.3399/BJGPO.2021.0189.

172. Swierad M, Dyrbus K, Szkodzinski J, Zembala MO, Kalarus Z, Gasior M. Telehealth visits in a tertiary cardiovascular center as a response of the healthcare system to the severe acute respiratory syndrome coronavirus 2 pandemic in Poland. Pol Arch Intern Med. 2020 Aug 27;130(7-8):700-3. PMID: 32426953. doi: 10.20452/pamw.15370.

173. Tensen E, Kuziemsky C, Jaspers MW, Peute LW. General Practitioners' Perspectives About Remote Dermatology Care During the COVID-19 Pandemic in the Netherlands: Questionnaire-Based Study. JMIR Dermatol. 2023 Jun 13;6:e46682. PMID: 37632975. doi: 10.2196/46682.

174. Testa S, Mayora-Ibarra O, Piras EM, Balagna O, Micocci S, Zanutto A, et al. Implementation of tele visit healthcare services triggered by the COVID-19 emergency: the Trentino Province experience. Z Gesundh Wiss. 2022;30(1):77-92. PMID: 34150467. doi: 10.1007/s10389-021-01609-8.

175. Thomas IS, L. Q. C.; Rutkowski, K. Synchronous Telemedicine in Allergy: Lessons Learned and Transformation of Care During the COVID-19 Pandemic. J ALLERGY CLIN IMMUNOL PRACT. 2020;9(1).

176. Tortajada-Goitia B, Morillo-Verdugo R, Margusino-Framiñán L, Marcos JA, Fernández-Llamazares CM. Survey on the situation of telepharmacy as applied to the outpatient care in hospital pharmacy departments in Spain during the COVID-19 pandemic. Farm Hosp. 2020 Jul 1;44(4):135-40. PMID: 32646343. doi: 10.7399/fh.11527.

177. Tyler JMBP, A. C.; Wooster, J.; Vasilakis, C.; Wood, R. M. The impact of increased outpatient telehealth during COVID‐19: Retrospective analysis of patient survey and routine activity data from a major healthcare system in England. Int J Health Plann Mgmt. 2021;36:1338–45.

178. van de Haar J, Hoes LR, Coles CE, Seamon K, Frohling S, Jager D, et al. Caring for patients with cancer in the COVID-19 era. Nat Med. 2020 May;26(5):665-71. PMID: 32405058. doi: 10.1038/s41591-020-0874-8.

179. van de Vijver S, Hummel D, van Dijk AH, Cox J, van Dijk O, Van den Broek N, et al. Evaluation of a Digital Self-management Platform for Patients With Chronic Illness in Primary Care: Qualitative Study of Stakeholders' Perspectives. JMIR Form Res. 2022 Aug 3;6(8):e38424. PMID: 35921145. doi: 10.2196/38424.

180. Vassallo CSH, A.; Abela, S. An Audit on the Provision of Telemedicine in Primary Care in Malta during the COVID-19. Malta Medical Journal. 2024;36(1).

181. Vasta R, Moglia C, D'Ovidio F, Di Pede F, De Mattei F, Cabras S, et al. Telemedicine for patients with amyotrophic lateral sclerosis during COVID-19 pandemic: an Italian ALS referral center experience. Amyotroph Lateral Scler Frontotemporal Degener. 2021 May;22(3-4):308-11. PMID: 32924624. doi: 10.1080/21678421.2020.1820043.

182. Viegas L, Dupie I, Rigal L, Van Poel E, Willems S, Beaupin A, et al. [Triage of patients and remote consultations in primary care facilities during the COVID-19 pandemic in France (PRICOV-19 study)]. Sante Publique. 2023 Dec 11;35(4):393-403. PMID: 38078634. doi: 10.3917/spub.234.0393.

183. Wanat M, Hoste M, Gobat N, Anastasaki M, Bohmer F, Chlabicz S, et al. Transformation of primary care during the COVID-19 pandemic: experiences of healthcare professionals in eight European countries. Br J Gen Pract. 2021 Aug;71(709):e634-e42. PMID: 33979303. doi: 10.3399/BJGP.2020.1112.

184. Wherton J, Greenhalgh T, Shaw SE. Expanding Video Consultation Services at Pace and Scale in Scotland During the COVID-19 Pandemic: National Mixed Methods Case Study. J Med Internet Res. 2021 Oct 7;23(10):e31374. PMID: 34516389. doi: 10.2196/31374.

185. Wilk M, Surowiec P, Matejko B, Wrobel A, Zieba-Parkitny J, Cyganek K, et al. Diabetes Management Delivery and Pregnancy Outcomes in Women with Gestational Diabetes Mellitus during the First Wave of the 2020 COVID-19 Pandemic: A Single-Reference Center Report. J Diabetes Res. 2021;2021:5515902. PMID: 34307689. doi: 10.1155/2021/5515902.

186. Willems LM, Balcik Y, Noda AH, Siebenbrodt K, Leimeister S, McCoy J, et al. SARS-CoV-2-related rapid reorganization of an epilepsy outpatient clinic from personal appointments to telemedicine services: A German single-center experience. Epilepsy Behav. 2020;112. doi: 10.1016/j.yebeh.2020.107483.

187. Wu F, Rotimi O, Laza-Cagigas R, Rampal T. The Feasibility and Effects of a Telehealth-Delivered Home-Based Prehabilitation Program for Cancer Patients during the Pandemic. Curr Oncol. 2021 Jun 17;28(3):2248-59. PMID: 34204531. doi: 10.3390/curroncol28030207.

188. Zakrzewski KM, Mularczyk-Tomczewska P, Koweszko T, Mosiolek A, Silczuk A. Telemedicine in polish primary care during and after the COVID-19 crisis: a retrospective analysis of over 720,000 consultations. Front Public Health. 2025;13:1695625. PMID: 41358208. doi: 10.3389/fpubh.2025.1695625.

189. Zondag AGM, Haitjema S, de Groot MCH, de Boer AR, van Solinge WW, Bots ML, et al. Comparison of outpatient attendance, cardiovascular risk management and cardiovascular health across preCOVID-19, during and postCOVID-19 periods: a prospective cohort study. BMJ Open. 2025 Jul 16;15(7):e092374. PMID: 40669897. doi: 10.1136/bmjopen-2024-092374.
